# Transcriptional profiling of an *Fd-GOGAT1/GLU1 *mutant in *Arabidopsis thaliana *reveals a multiple stress response and extensive reprogramming of the transcriptome

**DOI:** 10.1186/1471-2164-11-190

**Published:** 2010-03-22

**Authors:** Ralph Kissen, Per Winge, Diem Hong Thi Tran, Tommy S Jørstad, Trond R Størseth, Tone Christensen, Atle M Bones

**Affiliations:** 1 Department of Biology, Norwegian University of Science and Technology (NTNU), NO-7491 Trondheim, Norway; 2 SINTEF Fisheries and Aquaculture, NO-7465 Trondheim, Norway; 3 Current address: Scandpower AS, NO-7462 Trondheim, Norway; 4 Current address: Department of Cancer Research and Molecular Medicine, Norwegian University of Science and Technology (NTNU), NO-7489 Trondheim, Norway

## Abstract

**Background:**

Glutamate plays a central position in the synthesis of a variety of organic molecules in plants and is synthesised from nitrate through a series of enzymatic reactions. Glutamate synthases catalyse the last step in this pathway and two types are present in plants: NADH- or ferredoxin-dependent. Here we report a genome wide microarray analysis of the transcriptional reprogramming that occurs in leaves and roots of the *A. thaliana *mutant *glu1-2 *knocked-down in the expression of *Fd-GOGAT1 *(*GLU1*; *At5g04140*), one of the two genes of *A. thaliana *encoding ferredoxin-dependent glutamate synthase.

**Results:**

Transcriptional profiling of *glu1-2 *revealed extensive changes with the expression of more than 5500 genes significantly affected in leaves and nearly 700 in roots. Both genes involved in glutamate biosynthesis and transformation are affected, leading to changes in amino acid compositions as revealed by NMR metabolome analysis. An elevated glutamine level in the *glu1-2 *mutant was the most prominent of these changes. An unbiased analysis of the gene expression datasets allowed us to identify the pathways that constitute the secondary response of an *FdGOGAT1/GLU1 *knock-down. Among the most significantly affected pathways, photosynthesis, photorespiratory cycle and chlorophyll biosynthesis show an overall downregulation in *glu1-2 *leaves. This is in accordance with their slight chlorotic phenotype. Another characteristic of the *glu1-2 *transcriptional profile is the activation of multiple stress responses, mimicking cold, heat, drought and oxidative stress. The change in expression of genes involved in flavonoid biosynthesis is also revealed. The expression of a substantial number of genes encoding stress-related transcription factors, cytochrome P450 monooxygenases, glutathione S-transferases and UDP-glycosyltransferases is affected in the *glu1-2 *mutant. This may indicate an induction of the detoxification of secondary metabolites in the mutant.

**Conclusions:**

Analysis of the *glu1-2 *transcriptome reveals extensive changes in gene expression profiles revealing the importance of Fd-GOGAT1, and indirectly the central role of glutamate, in plant development. Besides the effect on genes involved in glutamate synthesis and transformation, the *glu1-2 *mutant transcriptome was characterised by an extensive secondary response including the downregulation of photosynthesis-related pathways and the induction of genes and pathways involved in the plant response to a multitude of stresses.

## Background

**Figure 1:**
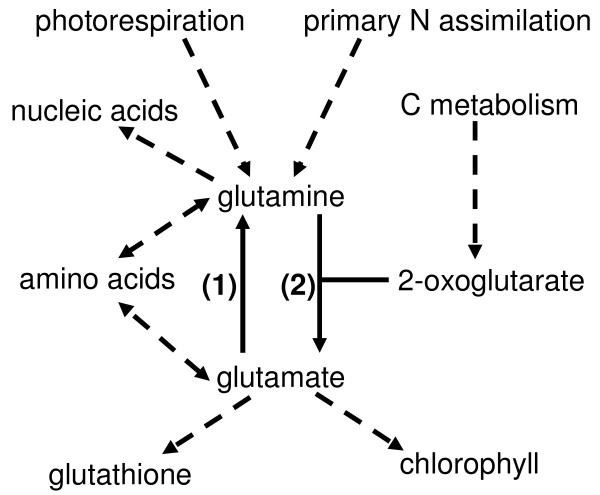
**The central role of glutamine/glutamate in plant metabolism**. The central role of glutamine/glutamate in plant metabolism is represented schematically. Plain arrows indicate the enzymatic reactions catalysed by glutamine synthetase (1) and glutamate synthase (2). Dashed arrows represent pathways that feed into or are affected by these two key amino acids. The representation is inspired by Forde and Lea [48].

Nitrogen is an important nutrient for plants and a limiting factor in plant development. It is mainly in the form of nitrate through nitrate transporters that nitrogen is taken up by plants. Nitrate is first reduced to nitrite and subsequently to ammonium through the action of nitrate reductase (EC 1.7.1.1) and nitrite reductase (EC 1.7.7.1), respectively. Glutamine synthetase (EC 6.3.2.1) catalyses the incorporation of this ammonium into glutamate and thereby producing glutamine. Subsequently, glutamate synthase transfers the amide amino group of glutamine to 2-oxoglutarate, yielding two molecules of glutamate. Production of glutamate is a key point in the synthesis of a variety of organic molecules, such as nucleic acids, amino acids and secondary metabolites (for review: [1]; Figure 1).

Besides the function in primary nitrogen assimilation, the GS/GOGAT pathway plays a central role in the reassimilation of ammonium produced by photorespiration (for review: [2]). Photorespiration is a photosynthesis-related pathway where O_2 _is taken up and CO_2 _is released due to the oxygenation of ribulose-1,5-biphosphate (RuBP) catalysed by RuBP carboxylase/oxygenase [3].

Plants possess two forms of glutamate synthase, which are both localized in plastids. One uses NADH as electron donor and is commonly called NADH-GOGAT (EC 1.4.1.14; GOGAT for "glutamine oxoglutarate aminotransferase"). The other one uses ferredoxin as electron donor and is called Fd-GOGAT (EC 1.4.7.1)(for review: [4]). In *Arabidopsis thaliana*, NADH-GOGAT is encoded by a single gene (*At5g53460*) whereas Fd-GOGAT is encoded by two genes, previously called *GLU1 *(*Fd-GOGAT1*, *At5g04140*) and *GLU2 *(*Fd-GOGAT2*, *At2g41220*)[5]. The two genes encoding Fd-GOGAT in *A. thaliana *show contrasting patterns of expression, with *Fd-GOGAT1 *expression being highest in leaves, whereas *Fd-GOGAT2 *is mostly expressed in roots [5,6]. Total glutamate synthase activity in *A. thaliana *is to a very large extent due to ferredoxin-dependent glutamate synthase, Fd-GOGAT1 contributing most [5,7]. *Fd-GOGAT1 *and *Fd-GOGAT2 *expression is also regulated differently: light causes a dramatic increase in *Fd-GOGAT1 *whereas *Fd-GOGAT2 *expression is not or only slightly affected. Similarly, *Fd-GOGAT1 *but not *Fd-GOGAT2 *expression is induced by sucrose [5]. Both Fd-GOGATs are localized to plastids but a mitochondrial localisation of Fd-GOGAT1 has also recently been shown [8].

Plants deficient in Fd-GOGAT activity have been described in *A. thaliana*, under several names (*gluS *[9], *gltS *[7], *gls *[5], *glu1 *[8]), and in other species such as barley and tobacco [10,11]. A chlorotic phenotype and a lethal phenotype under photorespiratory conditions, which indicate the importance of glutamate synthase in the respiratory pathway, are characteristic for Fd-GOGAT mutants [5,9].

The aim of the study was to characterise the transcriptional reprogramming that occurs in an *A. thaliana *mutant named *glu1-2*, knocked-down in the expression of *Fd-GOGAT1 *(*GLU1*; *At5g04140*) and to relate this to metabolic and phenotypic changes observed for this mutant.

We report here the genome wide transcriptional analysis by microarray and the metabolic profiling by NMR spectroscopy of *in vitro *grown *glu1-2 *mutant plantlets. These analyses identified substantial reprogramming of several pathways and processes in the mutant. These include primary and secondary nitrogen assimilation, leading to changes in the levels of certain amino acid, and photosynthesis related processes. The mutant was also affected in flavonoid biosynthesis and exhibited extensive transcriptional changes indicating the induction of multiple stress responses.

## Results and Discussion

### Phenotype of the *glu1-2 *mutant

**Figure 2:**
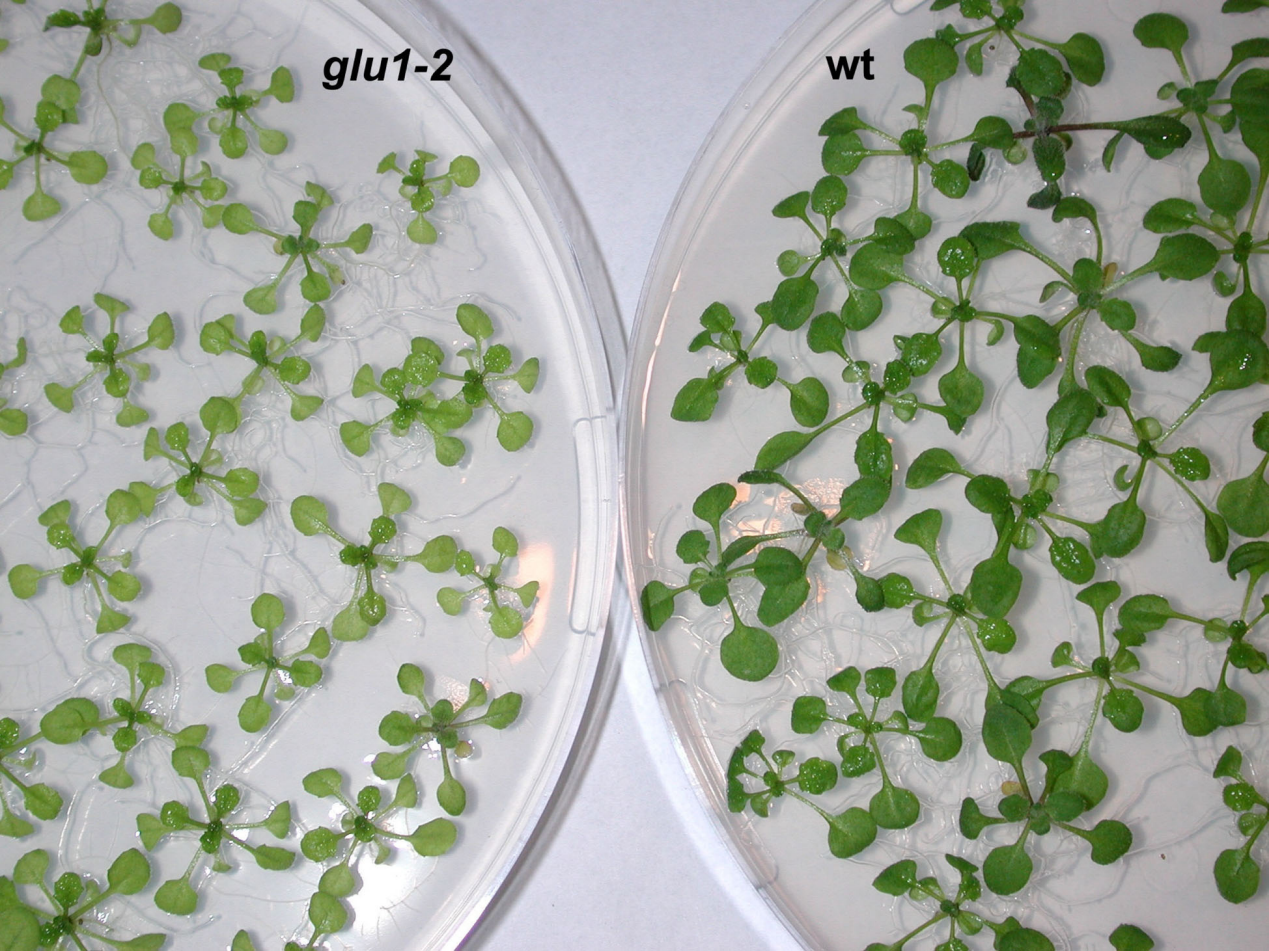
**Chlorotic phenotype of *glu1-2 *mutant plants**. Picture showing the chlorotic phenotype of eighteen-day old *glu1-2 *mutants (left) compared to Col-0 wild-type plants (right) grown *in vitro*.

An *A. thaliana *mutant presenting a T-DNA insertion in the *Fd-GOGAT1 *(*GLU1, GLS1; At5g04140*) gene was used in the present study. This mutant will hence be referred to as *glu1-2 *mutant hereafter. Under the *in vitro *growth conditions that were used in the present study, *glu1-2 *mutant plants exhibited a moderate chlorotic phenotype and reduced growth compared to wild-type Col-0 plants (Figure 2).

### Global overview and comparison of gene datasets that are affected in the *Fd-GOGAT1 *mutant leaves and roots

Changes in gene expression in leaves and roots of 18 day old *in vitro *grown *A. thaliana glu1-2 *plantlets were analysed using a genome wide microarray approach. This analysis showed that the expression of a high number of genes was affected in the *glu1-2 *mutant. Only genes whose expression was identified as being significantly changed at P = 0.01 were retained.

With 5615 genes whose expression were significantly affected (either induced or repressed) in leaves versus 687 genes in roots, there was an 8-fold difference in the number of affected genes between the two organs (Table 1). This was most likely due to the fact that *Fd-GOGAT1*, as opposed to *Fd-GOGAT2 *and *NADH-GOGAT*, has a much lower expression in roots than in leaves, which will be discussed later on. Some probes hybridizing to genes encoded by mitochondrial and chloroplastic genomes were found in the dataset of genes downregulated in leaves of the *glu1-2 *mutant. Within each organ the ratio between number of induced and the number of repressed genes was only slightly biased towards induction (1.09 in leaves and 1.27 in roots). When comparing the change fold in expression between the *glu1-2 *mutant and the wild-type, the log_2 _ratio of affected genes in leaves varied between 6.41 and -5.73 whereas that in roots was much more moderate with a variation between 1.71 and -2.61 (Additional file 1).

**Table 1:** Overview of genes differentially expressed between the *glu1-2 *mutant and the wild-type mutant

Organ	Change in expression	Number of genes
leaf	increased	2957

leaf	reduced	2708*

root	increased	384

root	reduced	303

Number of genes genes whose expression is significantly (P = 0.01) increased or reduced in the respective *glu1-2 *mutant organ versus that in wild-type. The details on the affected genes are given in Additional file 1.

*this number includes genes from mitochondrion and plastid genomes.

**Figure 3:**
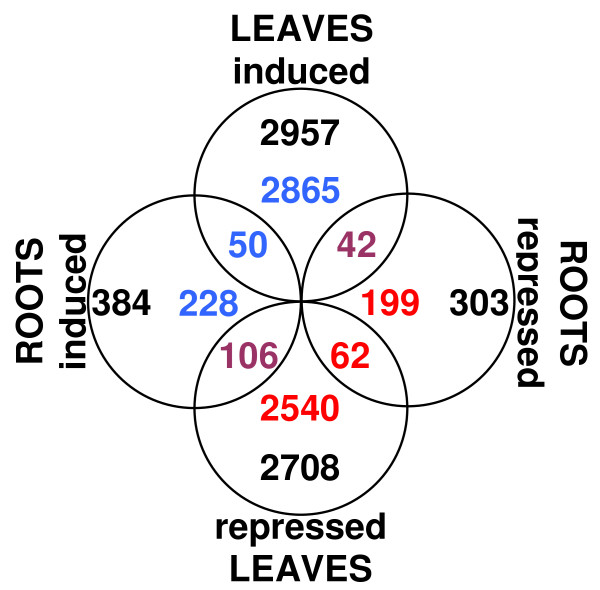
**Overview of the transcriptional changes in the *glu1-2 *mutant**. Venn diagram representing the genes that are significantly (P = 0.01) affected in the *glu1-2 *mutant versus wild-type plants. The total number of genes induced or repressed in leaves or roots are indicated in black. Induced genes are indicated in blue and repressed genes are indicated in red. Genes that are affected in both roots and leaves but show differential regulation are indicated in violet.

In *glu1-2 *mutant leaves, 2865 of the 2957 (almost 97%) induced genes were exclusively affected in this tissue (Figure 3). The 92 genes whose expression was also affected in roots, were almost equally distributed between induced (50) and repressed (42). genes. Among the 2708 genes repressed in leaves, 2540 (almost 94%) were only affected in leaves. Of the remaining 168 genes that are repressed in leaves and affected in roots, there is a clear bias towards induction. In *glu1-2 *roots, 228 out of 384 (over 59%) induced genes and 199 out of 303 (over 65%) repressed genes were exclusively affected in this tissue. Among the remaining genes, which were also affected in leaves, the induced ones were more prevalent than the repressed ones, irrespective of whether they were up-or down-regulated in leaves.

### Analysis of overrepresented gene ontologies and affected pathways among affected genes in the *glu1-2 *mutant indicates a reprogramming of several biological processes

**Figure 4:**
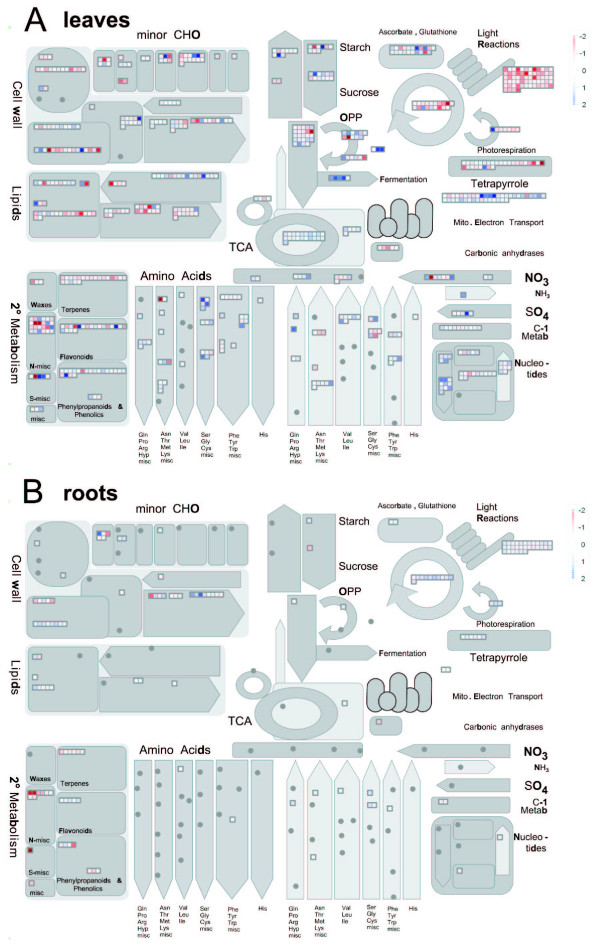
**Metabolic pathways covered by the transcriptional changes affecting the *glu1-2 *mutant**. Overview of expression changes in metabolic pathways in *glu1-2 *mutant leaves (**A**) and roots (**B**) using the MapMan software [12]. Represented are only the genes showing a significant (P = 0.01) change in expression between the *glu1-2 *mutant and the wild-type and that have been attributed to the respective bins by MapMan. Genes whose expression is increased or decreased in the *glu1-2 *mutant tissue versus the corresponding wild-type tissue are shown by an increasingly intense blue and red colour, respectively. The graduation can be seen on the scale that is represented in the top right corner of each subfigure. A change in expression of log_2 _= 2.0 scale was chosen as giving full saturation.

As seen above, a large number of genes are affected in the *glu1-2 *mutant, notably the leaves, which indicates that a profound transcriptional reprogramming takes place. The schematic representation by Mapman [12] illustrates to what extent different cellular processes and metabolic pathways are affected in the *glu1-2 *mutant (Figure 4). In order to identify the most relevant ones, an unbiased analysis of affected gene ontologies (GOs) and pathways was performed.

An analysis of overrepresented GO terms of the "biological process" classification using GOstat [13](P = 0.01 level with FDR/Benjamini correction) on the different *glu1-2 *transcriptional datasets was performed. The results of this analysis are shown in detail in Additional file 2 and summarised below.

When this analysis is applied to the genes whose expression is affected in *glu1-2 *leaves, not discriminating between induced and repressed ones, 54 overrepresented GO_biological_process terms are identified. When the datasets are separated into genes induced or repressed in *glu1-2 *leaves, 73 and 19 overrepresented GO terms are identified respectively. In comparison, only 13 GO_biological_process terms are overrepresented among the affected (induced or repressed) genes in *glu1-2 *roots. Analysis on *glu1-2 *root-induced and -repressed genes separately identifies 34 and 0 overrepresented GO_biological_process terms, respectively. When combining expression patterns from leaves and roots 2 GO_biological_process terms are overrepresented among affected genes, while the subset of genes downregulated in leaves and upregulated in roots reveals three additional overrepresented GO_biological_processes. Two terms are overrepresented among genes induced in both organs of the *glu1-2 *mutant (Additional file 2).

Hence, GOstat identifies in total 124 unique GO_biological_process terms (confounded levels) that are overrepresented in the *glu1-2 *transcriptional profile changes (Additional file 2). Analysis with two further algorithms, PathExpress [14] and GeneBins [15], gave overlapping results to the ones obtained by GOstat as to which biological processes and pathways are affected in the *glu1-2 *mutant. These results are therefore not further detailed in the text but are shown in Additional files 3 and 4.

**Figure 5:**
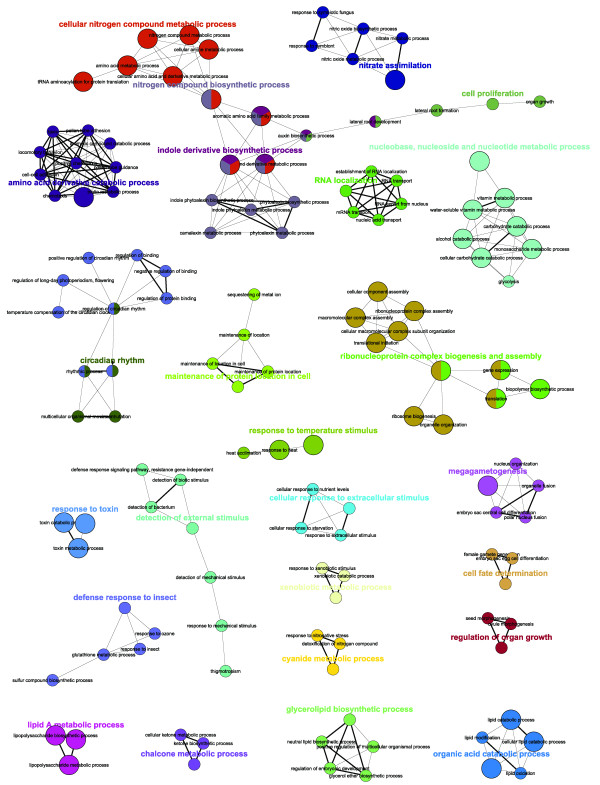
**Network representations of enriched GO categories amongst genes induced in *glu1-2 *mutant leaves**. Representations generated by ClueGO [17] of functionally grouped networks of enriched GO categories among genes whose expression is induced in leaves of the *glu1-2 *mutant compared to leaves of the wild-type. GO terms are represented as nodes based on their kappa score level (≥ 0.3), only networks with at least three nodes being represented. The node size represents the term enrichment significance. The label of the most significant term is used as leading group term.

**Figure 6:**
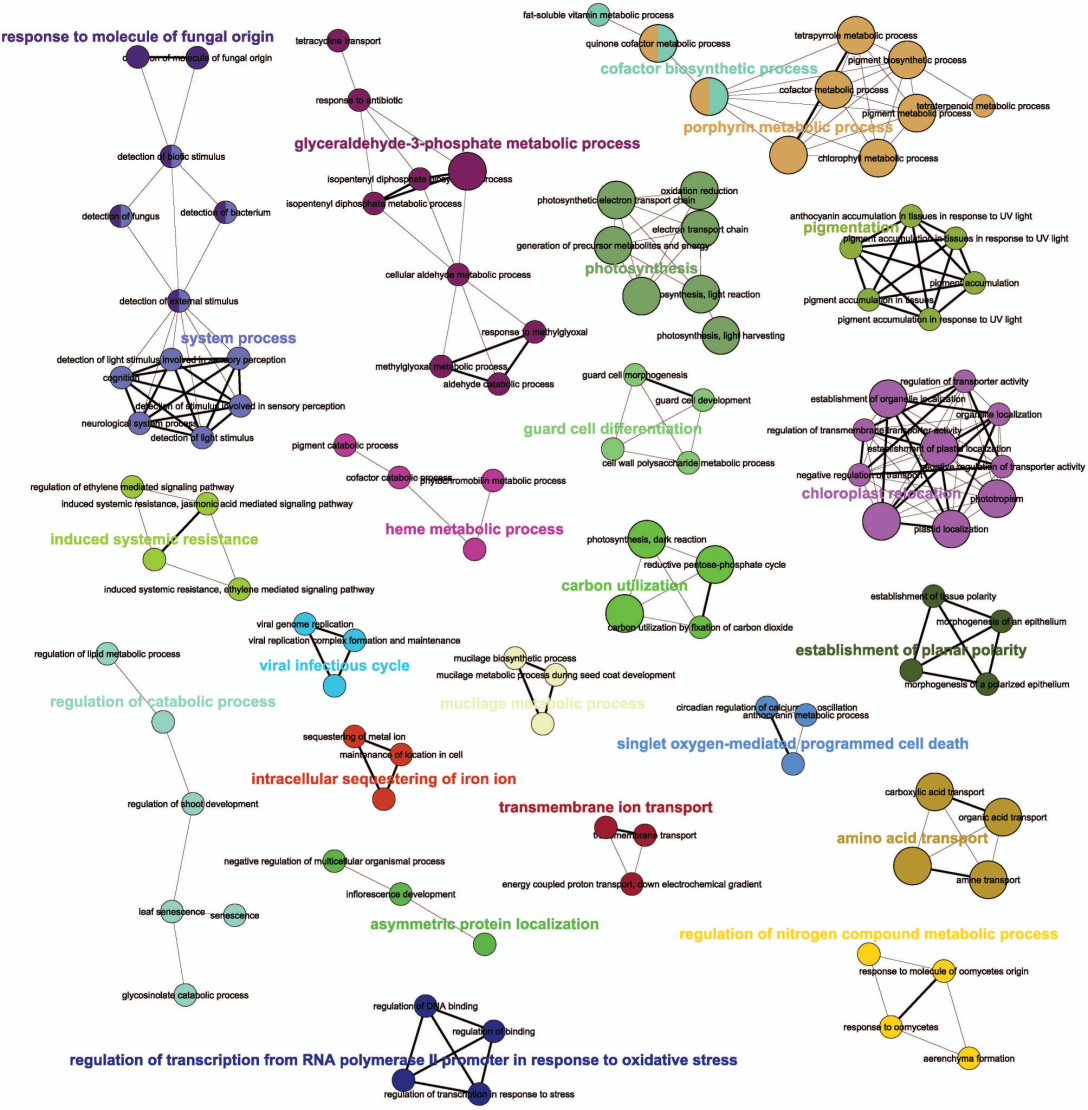
**Network representations of enriched GO categories amongst genes repressed in *glu1-2 *mutant leaves**. Representations generated by ClueGO [17] of functionally grouped networks of enriched GO categories among genes whose expression is repressed in leaves of the *glu1-2 *mutant compared to leaves of the wild-type. GO terms are represented as nodes based on their kappa score level (≥ 0.3), only networks with at least three nodes being represented. The node size represents the term enrichment significance. The label of the most significant term is used as leading group term.

**Figure 7:**
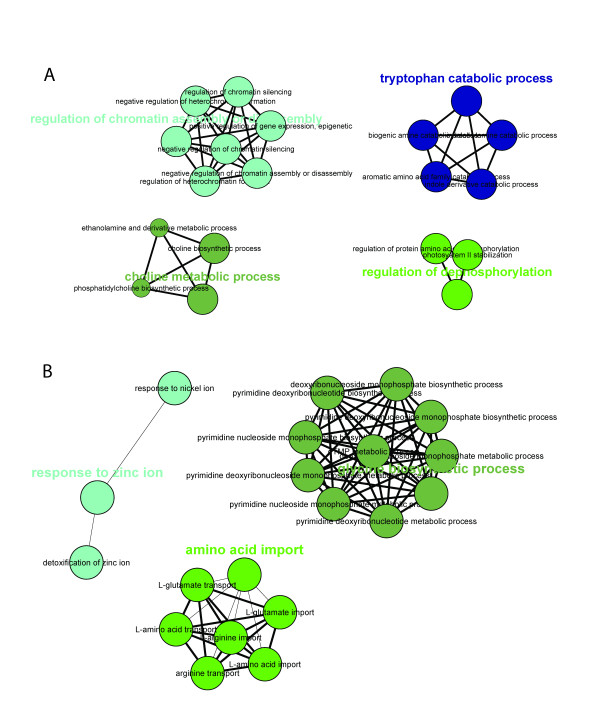
**Network representations of enriched GO categories amongst genes affected in *glu1-2 *mutant roots**. Representations generated by ClueGO [17] of functionally grouped networks of enriched GO categories among genes whose expression is induced (A) or repressed (B) in roots of the *glu1-2 *mutant compared to roots of the wild-type. GO terms are represented as nodes based on their kappa score level (≥ 0.3), only networks with at least three nodes being represented. The node size represents the term enrichment significance. The label of the most significant term is used as leading group term.

Visualisation of the networks of GO terms that are enriched in the *glu1-2 *mutant versus wild-type, using the Cytoscape [16] plug-in ClueGO [17], illustrates the complexity of the transcriptional response in *glu1-2 *mutant leaves (Figures 5 and 6). Notably, among genes upreglated in *glu1-2 *leaves GO terms related to metabolic processes of nitrogen and carbohydrate compounds (Figure 5), the regulation of transcription and the response to a diversity of stimuli are overrepresented. GO categories related to the latter two are also overrepresented among downregulated genes in *glu1-2 *leaves, in addition to photosynthesis and pigment/porphyrin biosynthesis (Figure 6). Although the complexity of GO-term networks enriched in the *glu1-2 *mutant roots versus wild-type roots is reduced (Figure 7), the T-DNA insertion in *Fd-GOGAT1 *has nevertheless a considerable impact on the root transcriptome as evidenced by the number of affected genes (i.e. 687) and the affected GO-terms (Additional files 2 to 4).

The large number of genes with modified expression and the analyses described above indicate that a large number of pathways and processes are seemingly affected in the *glu1-2 *mutant. Interestingly, glutamate biosynthesis and nitrogen metabolism are not often identified as such, and if so are indicated as induced in leaves. However as these pathways most likely constitute the plant's primary response to the knock-down of *Fd-GOGAT1*, the expression data of genes involved in these pathways will be analysed in a first part. In a second part the focus will be put on some of the processes that, despite the differences in algorithms and category definitions and terms, are recurrently identified by the performed analyses. These processes most likely constitute a secondary response of the mutant plant and encompass photosynthesis and related processes as well as aspects of a multiple stress response.

Even within the selected pathways and processes that will be presented below it is out of scope to present and discuss all aspects in the text. The reader is therefore kindly referred to the respective Additional files containing the detailed information about affected genes.

### Analysis of genes involved in glutamate biosynthesis or related pathways whose expression is affected in leaves or roots of the *glu1-2 *mutant

#### Changes in expression levels of genes involved in glutamate biosynthesis and related pathways

In order to show how glutamate metabolism and related pathways are affected in the *glu1-2 *mutant, an overview of the major genes and enzymes involved (Table 2) is given below, starting from the endpoint (i.e. glutamine and glutamate synthesis) and expanding to the steps leading up to glutamate, before focussing on the pathways that utilize glutamate in the production of other compounds. This is complemented by analysis of certain amino acid contents in the *glu1-2 *mutant.

**Table 2:** Genes that are involved in major glutamate-related pathways and that are differentially expressed between the *glu1-2 *mutant and the wild-type mutant

EC #	Enzyme/protein	Abbreviation	Gene ID	log_2 _root	log_2 _leaf
1.4.1.7	Fd-dependent glutamate synthase	Fd-GOGAT 1/GLU1	*AT5G04140*		-5.738

1.4.1.7	Fd-dependent glutamate synthase	Fd-GOGAT 2/GLU2	*AT2G41220*		1.070

1.4.1.14	NADH-dependent glutamate synthase	NADH-GOGAT	*AT5G53460*		0.712

1.7.1.1	nitrate reductase	NIA2/NR2	*AT1G37130*		0.438

1.7.7.1	nitrite reductase	NIR1	*AT2G15620*		0.687

6.3.1.2	glutamine synthetase	GLN1.1	*AT5G37600*		1.296

6.3.1.2	glutamine synthetase	GLN1.3	*AT3G17820*		0.669

6.3.1.2	glutamine synthetase	GLN1.4	*AT5G16570*		-0.580

6.3.5.4	asparagine synthetase	ASN1	*AT3G47340*		-2.913

6.3.5.4	asparagine synthetase	ASN2	*AT5G65010*		-0.486

3.5.1.1	asparaginase		*AT3G16150*		-0.481

2.6.1.1	aspartate aminotransferase	ASP1	*AT2G30970*		0.582

2.6.1.1	aspartate aminotransferase	ASP2	*AT5G19550*		1.132

2.6.1.1	aspartate aminotransferase	ASP3	*AT5G11520*		0.709

2.6.1.1	aspartate aminotransferase	ASP4	*AT1G62800*		0.354

2.6.1.2	alanine:2-oxoglutarate aminotransferase	AlaAT1 AlaAT2	*AT1G17290* *AT1G72330*		0.438

2.6.1.2/2.6.1.4	glutamate:glyoxylate aminotransferase	GGAT2	*AT1G70580*		-0.680

	glycine decarboxylase complex -- H protein	AtGDH3	*AT1G32470*	0.593	

	glycine decarboxylase complex -- H protein	AtGDH1	*AT2G35370*	0.768	

1.8.1.4	glycine decarboxylase complex -- L protein	AtmLPD1	*AT3G17240*		0.689

1.4.4.2	glycine decarboxylase complex -- P protein	AtGLDP1	*AT4G33010*	0.439	-1.126

1.4.4.2	glycine decarboxylase complex -- P protein	AtGLDP2	*AT2G26080*		-0.532

2.1.2.10	glycine decarboxylase complex -- T protein	AtGDT1	*AT1G11860*		-0.600

2.1.2.1	serine hydroxymethyltransferase	SHM1	*AT4G37930*		-0.513

2.1.2.1	serine hydroxymethyltransferase	SHM3	*AT4G32520*		0.532

2.1.2.1	serine hydroxymethyltransferase	SHM4 SHM5	*AT4G13930* *AT4G13890*		0.535

4.1.1.15	glutamate decarboxylase	GAD3 GAD4	*AT2G02000* *AT2G02010*		1.441

2.6.1.19	γ-aminobutyric acid transaminase	GABA-T1	*AT3G22200*		0.720

1.2.1.24	succinic semialdehyde dehydrogensase	SSADH1	*AT1G79440*		0.516

1.1.1.86	ketolacid reductoisomerase	KARI	*At3g58610*		0.380

4.2.1.9	dehydroxyacid dehydratase	DHAD	*At3g23940*		0.857

6.3.2.2	glutamate-cysteine ligase	GSH1	*AT4G23100*		0.587

6.3.2.3	glutathione synthetase	GSH2	*AT5G27380*		0.855

1.4.1.3	glutamate dehydrogenase	GDH2	*AT5G07440*		1.126

2.3.1.1	N-acetyltransferase	NAGS	*AT4G37670*		0.755

6.3.5.5	carbamoyl-phosphate synthase	CPS	*AT3G27740*		0.696

6.3.5.5	carbamoyl-phosphate synthase	CPS	*AT1G29900*		0.701

2.1.3.3	ornithine carbamoyltransferase	OTC	*AT1G75330*		0.436

6.3.4.5	arginosuccinate synthase	AS	*AT4G24830*		0.596

	nitrate transporter	NRT1.1	*AT1G12110*		0.432

	nitrate transporter	NRT1.3/NTP3	*AT3G21670*		-0.703

	nitrate transporter	NRT1.5	*AT1G32450*		0.382

	nitrate transporter	NRT1.7	*AT1G69870*	0.501	1.760

	nitrate transporter	AtNRT2.7	*AT5G14570*		0.546

	nitrate uptake	NRT3.1/NAR2.1	*AT5G50200*		0.939

	nitrate/proton antiporter	AtCLCa	*AT5G40890*		-0.689

	ammonium transporter	AMT2.1	*AT2G38290*		1.495

	vacuolar tonoplast intrinsic protein	AtTIP2;1	*AT3G16240*		-0.554

	vacuolar tonoplast intrinsic protein	AtTIP2;3	*AT5G47450*	-1.105	

	amino acid permease	AAP1	*AT1G58360*		-0.523

	amino acid permease	AAP4	*AT5G63850*	-0.586	-0.470

	amino acid permease	AAP5	*AT1G44100*		0.901

	amino acid permease	AAP6	*AT5G49630*		-0.532

	glutamate influx	LHT1	*AT5G40780*	-0.411	

	glutamate receptor family protein	AtGLR1.4	*AT3G07520*		-0.453

	glutamate receptor family protein	AtGLR3.2	*AT4G35290*		-0.613

	glutamate receptor family protein	AtGLR3.7	*AT2G32400*		0.338

**Figure 8:**
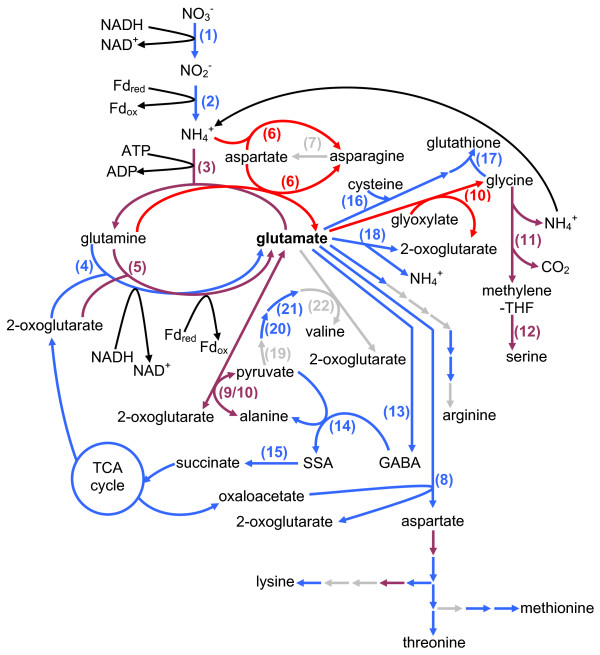
**Primary nitrogen assimilation and glutamate metabolism**. Simplified schematic representation of the pathways leading to the synthesis and the conversion of glutamate and the way they are affected in the *glu1-2 *mutant. Only the data related to *glu1-2 *leaves is depicted. Colour code: blue are upregulated, red are downregulated, violet are both up- and downregulated steps whereas grey are unaffected steps of these pathways. Enzymes: (1) nitrate reductase, (2) nitrite reductase, (3) glutamine synthetase, (4) NADH-GOGAT, (5) Fd-GOGAT, (6) asparagine synthase, (7) asparaginase, (8) aspartate aminotransferase, (9) alanine:2-oxoglutarate aminotransferase, (10) glutamate:glyoxylate aminotransferase, (11) glycine decarboxylase complex, (12) serine hydroxymethyltransferase, (13) glutamate decarboxylase, (14) γ-aminobutyric acid transaminase, (15) succinic semialdehyde dehydrogenase, (16) glutamate-cysteine ligase, (17) glutathione synthetase, (18) glutamate dehydrogenase, (19) acetohydroxyacid synthase, (20) ketolacid reductoisomerase, (21) dehydroxyacid dehydratase, (22) branched-chain aminotransferase. Subcellular compartmentalisation is not taken into account in this figure and reductants are only indicated for some of the reactions. The detailed data of the genes depicted in this figure and related data for genes affected in *glu1-2 *roots is presented in Table 2.

List of genes encoding enzymes involved in N-assimilation and glutamate biosynthesis and transformation described in the text, and whose expression is affected by knocking down *Fd-GOGAT1 *in the *glu1-2 *mutant. Most of the listed enzymes are also depicted in Figure 8. The presence of two gene IDs in some boxes indicates a nondiscriminating probe, i.e. a probe recognising more than one transcript so that a change in expression can not be attributed to a specific gene.

#### Changes in expression levels of genes encoding glutamate synthases and glutamine synthetases

Comparison of expression levels in our microarray assays indicates that *Fd-GOGAT1 *(*GLU1*;*At5g04140*) is more highly expressed in leaves than in roots (signal intensity: log_2 _= 10.84 versus log_2 _= 5.06; Δlog_2 _= 5.78) of 18 day old *in vitro *grown *A. thaliana *wild type plants (Col-0 ecotype) (Additional file 1). This is in accordance with earlier published results and gene expression data publicly available [5,6,18,19]. As expected, the expression of *Fd-GOGAT1 *(*At5g04140*) was downregulated in the *glu1-2 *mutant (Table 2). There was a log_2 _ratio of -5.74 between the expression levels of *Fd-GOGAT1 *in leaves of wild type and the mutant. In roots the difference was more moderate with a reduction in expression of log_2 _= -0.54 (only significant at P = 0.05). It should however be noted that residual levels of transcripts for *Fd-GOGAT1 *are still detectable on the microarrays from the *glu1-2 *mutant, and that these are similar in roots and leaves.

The gene *GLU2 *(*At2g41220*) encoding Fd-GOGAT2, the second *A. thaliana *Fd-dependent glutamate synthase, is upregulated (log_2 _= 1.07) in leaves but not affected in roots of the *glu1-2 *mutant. The NADH-GOGAT (EC1.4.1.14) encoding gene *At5g53460 *is also slightly upregulated (log_2 _= 0.71) in *glu1-2 *leaves but not affected in roots (Table 2). This could indicate a partial recovery of the loss of the plastid-localized Fd-GOGAT1 by these enzymes in leaves as NADH-GOGAT and Fd-GOGAT2 are localized to plastids. *Fd-GOGAT2 *has however higher expression levels in roots than in leaves and is therefore more likely involved in primary nitrogen assimilation in roots [5]. In addition, NADH-dependent glutamate synthase activity only makes up a small percentage of the total glutamate synthase activity in *A. thaliana *leaves and NADH-dependent activity is not affected in Fd-GOGAT deficient mutants [7,9]. It should also be noted that posttranscriptional regulation has been hypothesised for Fd-GOGAT in tobacco and *A. thaliana *[20,21].

Glutamine is the substrate of GOGATs for the synthesis of glutamate (Figure 8) and glutamine synthetases (EC 6.3.1.2) catalyse the synthesis of glutamine from ammonium (NH_4_^+^) using ATP. Of the five putative *A. thaliana *genes encoding the cytoplasmic glutamine synthetase 1 (GS1), two were upregulated (*At5g37600*/*GLN1.1 *and *At3g17820*/*GLN1.3*) whereas one (*At5g16570*/*GLN1.4*) was slightly downregulated in *glu1-2 *leaves. The gene *At5g35630 *encoding glutamine synthetase 2 (GS2), which is dual-targeted to the plastid and mitochondria [22], was not affected. None of the genes encoding glutamine synthetases 1 or 2 were affected in the roots of the *glu1-2 *mutant.

#### Genes encoding enzymes involved in the primary nitrogen assimilation leading to the formation of glutamine and glutamate

Glutamine and glutamate play a central role in the primary assimilation of nitrogen (Figure 1). Hence, genes encoding enzymes catalysing the reactions leading to the formation of glutamine from nitrate are affected in the *glu1-2 *mutant (Table 2), mostly in leaves.

Some of the genes encoding nitrate reductase (NADH-NAR; EC 1.7.1.1) and nitrite reductase (NIR1; EC 1.7.7.1), are upregulated in *glu1-2 *leaves while unaffected in *glu1-2 *roots. These enzymes are responsible for the reduction of nitrate and nitrite respectively, leading to the formation of NH_4_^+^, which serves as substrate by glutamine synthetase. The upregulation of nitrate reductase expression levels was previously observed in tobacco plants deficient in Fd-GOGAT activity [23].

Glutamine and aspartate are used as substrates for the synthesis of glutamate and asparagine, in an ATP-dependent reaction catalysed by asparagine synthase (ASN; EC 6.3.5.4)(for review: [24]). Of the three *A. thaliana *genes encoding asparagine synthases, two were downregulated in *glu1-2 *leaves but none was affected in roots (Table 2). *ASN1 *(*At3g47340*) is one of the ten most downregulated genes in the *glu1-2 *mutant leaves. It has been shown that *ASN1 *is induced by dark and reduced by light (or sucrose) while *ASN2*, which is moderately downregulated, is induced by light. These two genes also respond differently to asparagine, glutamine and glutamate with *ASN1 *expression being induced and *ASN2 *expression being reduced ([24] and references therein). Overexpressing ASN1 leads to higher asparagine levels in seeds and phloem [25] and Masclaux-Daubresse et al. [21] have recently shown that asparagine synthase can also catalyse the formation of asparagine from aspartate using ammonium directly. None of the four genes encoding asparaginases (EC 3.5.1.1), which are responsible of degrading asparagine into aspartate, were affected in the leaves of the *glu1-2 *mutant (Figure 8). Hence, the reduced expression of asparagine synthase encoding genes in *glu1-2 *leaves could indicate lower levels of asparagine. In Fd-GOGAT deficient barley plants, the levels of asparagine are however higher than in wild-type plants [10].

### Multiple pathways leading to the transformation and degradation of glutamate are also affected

Glutamate serves directly or indirectly as substrate in the production of a series of compounds, like amino acids, nucleic acids, ureides, and polyamines (Figure 1; [26]). Hence, reducing *Fd-GOGAT1 *expression could have a knock-on effect on the genes encoding enzymes implicated in these various biosynthetic pathways (Figure 8).

#### Amino acid biosynthesis

Aspartate aminotransferases (ASPs or AATs; EC 2.6.1.1) catalyse the transfer of the α-amino group of glutamate to oxaloacetate to form aspartate and 2-oxoglutarate. In *glu1-2 *leaves, four of the five *A. thaliana *genes (putatively) encoding ASPs [26] are upregulated in *glu1-2 *leaves. Aspartate is a precursor of asparagine and the aspartate family of amino acids such as lysine, threonine and methionine (Figure 8). From the changes in expression levels of genes involved in these pathways the synthesis of these latter amino acids seems to be induced in *glu1-2 *leaves.

The transfer of the α-amino group of glutamate to pyruvate to form alanine is catalysed by alanine aminotransferases (AlaAT or AOAT; EC 2.6.1.2) which comprise four members, subdivided into two groups, in *A. thaliana *[27]. The first group, composed of AlaAT1 and AlaAT2 that possess alanine aminotransferase activity, is slightly induced in leaves but not in roots of the *glu1-2 *mutant (Table 2). AlaAT1 has recently been suggested to catalyse the reverse reaction (i.e. conversion of alanine to pyruvate) [28], which could lead to a production of glutamate (Figure 8) to compensate for the lack of Fd-GOGAT1. Low levels of glutamate may shift this reaction equilibrium to favour glutamate production. The members of the second group, two peroxisomal enzymes, possess a glycine (or glutamate:glyoxylate) aminotransferase (GGAT) activity in addition to their alanine aminotransferase activity (Figure 8). Glyoxylate is thereby transaminated to glycine, accompanied by the consumption of glutamate and the production of 2-oxoglutarate, during photorespiration. Neither of these two genes is affected in *glu1-2 *roots (Table 2) but the expression of *GGAT2 *(*At1g70580*) is reduced in *glu1-2 *leaves. The further conversion of glycine to serine involves the glycine decarboxylase complex (GDC) and serine hydroxymethyltransferase (SHMT; EC 2.1.2.1). The glycine decarboxylase complex is composed of four mitochondrial proteins (H, L, P and T) encoded by a total of eight genes in *A. thaliana *[29]. The genes encoding the two P proteins are both downregulated (Table 2). Knocking out these genes simultaneously provokes a lethal phenotype, also under nonphotorespiratory conditions, which points towards a role of GDC in other metabolic processes than photorespiration [30]. Of the seven *A. thaliana *genes putatively encoding serine hydroxymethyltransferases, four show a changed expression in *glu1-2 *leaves (Table 2). *SHM1 *(*At4g37930*) encoding the mitochondrial SHMT1 is downregulated. Interestingly, the knock-out mutant *shm1-1 *also displays a lethal photorespiratory phenotype [31] and a physical interaction between Fd-GOGAT1 and SHMT1 in mitochondria was recently established [8]. Three genes encoding putative cytosolic serine hydroxymethyltransferases were on the other hand almost equally upregulated in leaves. None of the SHMT-encoding genes was affected in *glu1-2 *roots (Table 2).

Arginine is formed from glutamate and glutamine in a multiple-reaction pathway [32] that shows a slight overall induction in *glu1-2 *mutant leaves (Figure 8; Table 2). This may be responsible for increased production of arginine that has been observed in Fd-GOGAT deficient plants and which may prevent excessive accumulation of glutamine [11]. In root tissue of the *glu1-2 *mutant none of the transcripts of these genes involved in arginine synthesis were affected.

Valine biosynthesis starts with the condensation of two molecules of pyruvate by acetohydroxyacid synthase (AHAS; EC 2.2.1.6), also known as acetolactate synthase [33]. Neither the gene *At3g48560 *encoding the catalytic subunit, nor the genes *At2g31810 *and *At5g16290 *encoding the regulatory subunit, are significantly affected in the *glu1-2 *mutant (Figure 8). The resulting 2-acetolactate is converted to 2,3-dihydroxy-3-isovalerate by ketolacid reductoisomerase (KARI; EC 1.1.1.86) and its gene *At3g58610 *is slightly upregulated in *glu1-2 *leaves. The gene *At3g23940 *encoding dehydroxyacid dehydratase (DHAD; EC 4.2.1.9), which catalyses the conversion of 2,3-dihydroxy-3-isovalerate to α-ketoisovalerate, is also induced in *glu1-2 *leaves. Branched-chain aminotransferases (BCAT; EC 2.6.1.42) catalyse the subsequent and last step in the synthesis of valine from α-ketoisovalerate, which is accompanied by the conversion of glutamate to 2-oxoglutarate. However none of the BCAT-encoding genes (*At1g10070*, *At3g49680 *and *At5g65780*) implicated in this step is affected in the *glu1-2 *mutant. Hence, a few of the genes encoding biosynthetic enzymes involved in valine biosynthesis are induced, indicating possibly a slight activation of the valine biosynthetic pathway.

#### Glutathione biosynthesis

Glutamate is used for the synthesis of glutathione in a two-reaction pathway (Figure 8) catalysed by glutamate-cysteine ligase (GSH1; EC 6.3.2.2) and glutathione synthetase (GSH2; EC 6.3.2.3). Both GSH1 (*At4g23100*) and GSH2 (*At5g27380*) are upregulated in *glu1-2 *mutant leaves (Table 2). Mutants deficient in GSH1 have been shown to contain lower levels of glutathione and are more sensitive to stresses. Complete knock-out of *GSH1 *leads to an embryo-lethal phenotype [34]. Upregulation of glutathione biosynthetic genes in *glu1-2 *may be connected to the upregulation of numerous glutathione S-transferases (see later).

#### Gamma-aminobutyrate and succinate synthesis

Under certain conditions glutamate may be converted to gamma-aminobutyrate (GABA) in a cytosolic reaction catalysed by glutamate decarboxylase (GAD; EC 4.1.1.15). The two GAD-encoding genes *GAD1 *(*At5g17330*) and *GAD2 *(*At1g65960*) initially identified in *A. thaliana *[35,36] are not affected in the leaves or roots of the *glu1-2 *mutant. Of the three additional genes encoding putative GADs that have recently been identified based on homology [37], GAD3 and/or GAD4 (undifferentiating probe) were upregulated in *glu1-2 *mutant leaves (Table 2). The gene encoding GABA-T1 (EC 2.6.1.19; *At3g22200*), the γ-aminobutyric acid transaminase that catalyses the conversion of GABA into succinic semialdehyde (SSA), simultaneously producing alanine from pyruvate, is also induced in leaves. The conversion of SSA to succinate by succinic semialdehyde dehydrogensase (SSADH) is also upregulated in *glu1-2 *leaves (Table 2).

#### Glutamate catabolism

Glutamate is catabolised into oxoglutarate and ammonium by glutamate dehydrogenase (GDH; EC 1.4.1.2) (Figure 8), a mitochondrial enzyme that exists under the form of homo- or heterohexamers of two subunits in *A. thaliana *[38]. Only *GDH2 *(*At5g07440*), encoding the β-subunit, is affected in the *glu1-2 *mutant, showing surprisingly an increased expression in mutant leaves (Table 2). Although the role of glutamate dehydrogenase in glutamate metabolism has remained controversial for a long time, recent evidence indicates that GDHs are indeed responsible for the deamination of glutamate leading to the formation of ammonium and 2-oxoglutarate [21,39]. Induction of GDH2 expression could hence lead to a further depletion of the pool of glutamate in the *glu1-2 *mutant but would simultaneously increase the levels of oxoglutarate to fuel the TCA cycle. Although the physiological role of GDH is currently still unclear, there seems to be a consensus that GDH is not essential for primary nitrogen assimilation. Instead a role of GDH in the breakdown of several amino acids into their corresponding keto-acids under carbon deficiency was proposed [39]. The the α- and β-subunit composition of glutamate dehydrogenase hexamers may also influence its activity and hence its physiological role [40]. Lancien et al. [41] proposed that ammonium and glutamine would favour the amination reaction. In tobacco plants with reduced Fd-GOGAT activity the aminating, but not the deaminating activity, of glutamate dehydrogenase was indeed reported [42].

#### Cellular uptake of glutamate

Amino acids can be exported from their site of synthesis and transported via the vascular tissue to newly developed tissues. In *A. thaliana*, glutamate is one of the predominant amino acids found in the phloem sap and xylem exudates [18]. Members of the *A. thaliana *amino acid permease (AAP) family have been shown to catalyse the low affinity influx of a broad range of amino acids, including glutamate [43]. Of these, *AAP1 *(*At1g58360*), *AAP4 *(*At5g63850*) and *AAP6 *(*At5g49630*) were moderately downregulated, whereas *AAP5 *(*At1g44100*) was induced in *glu1-2 *leaves (Additional file 1). AAP1 has recently been shown to be involved in glutamate uptake into root cells and may have a role in the efficient use of nitrogen resources in the rhizosphere [44], but its expression was not affected in *glu1-2 *roots. Only AAP4 (*At5g63850*) was affected in roots, with a lower expression in the mutant than in the wild-type.

The related *LHT1 *(*At5g40780*) gene which appears to encode a high-affinity glutamate influx system [45] was also moderately downregulated in *glu1-2 *roots but not affected in *glu1-2 *leaves. A role for LHT1 in root uptake of certain amino acids was also recently proposed, but its role in glutamate uptake may be limited as this was not affected in an *lht1 *mutant [46].

The gene *At2g01170 *encoding the recently identified bidirectional amino acid transporter BAT1 capable of import and export of glutamate and potentially involved in amino acid export from phloem to sink tissue [47] is moderately induced in *glu1-2 *leaves and not affected in roots.

#### Changes in expression levels og genes involved in the synthesis of 2-oxoglutarate and the TCA cycle

The synthesis of glutamate by NADH- and Fd-GOGATs necessitates 2-oxoglutarate, which is thus situated at the interface between C and N metabolism. As it is possible to affect the glutamate pool by feeding 2-oxoglutarate to plants, the supply of 2-oxoglutarate may be a key regulator of glutamate levels [48].

2-oxoglutarate is produced through the TCA cycle (for review: [41]) and several genes involved in the TCA cycle were upregulated in *glu1-2 *leaves (Figure 8; Additional file 5). These include genes encoding citrate synthase (CSY; EC2.3.3.1), succinate dehydrogenases (SDH; EC 1.3.5.1) and succinyl-CoA ligase (EC 6.2.1.4). Three of the five genes encoding NAD-dependent isocitrate dehydrogenase (IDH; EC 1.1.1.41) subunits are also upregulated in *glu1-2 *mutant leaves (Figure 8; Additional file 5). This increase in IDH transcript levels in the *glu1-2 *mutant is in contrast to the situation observed in tobacco plants with reduced Fd-GOGAT activity, where no change in transcript levels was observed. These tobacco plants did however show increased isocitrate dehydrogenase activity [42]. Recently, Lemaitre et al. [49] have shown that mutants lacking one of the three IDH subunits mentioned above do not exhibit changed levels in 2-oxoglutarate, glutamine and glutamate. The *glu1-2 *mutant leaves also showed an increase in the expression of four genes encoding putative components (EC 2.3.1.61 and EC 1.2.4.2) of the 2-oxoglutarate dehydrogenase system. On the other hand, the genes *At2g47510*/*At5g50950 *encoding fumarate hydratases (FUM; EC 4.2.1.2) were downregulated in *glu1-2 *leaves. Interestingly, none of the TCA cycle-implicated genes mentioned above were significantly affected in *glu1-2 *roots. These data indicate an overall induction of the TCA cycle in *glu1-2 *leaves potentially leading to an increase in 2-oxoglutarate production. Higher levels of 2-oxoglutarate have been observed in Fd-GOGAT deficient tobacco plants [11].

2-oxoglutarate can also be synthesised in the cytosol by export of citrate from mitochondria and the subsequent action of aconitases and isocitrate dehydrogenases. Two aconitase-encoding genes (ACO; EC 4.1.2.3) and two genes putatively encoding cytosolic NADP-dependent isocitrate dehydrogenases (IDCH; EC 1.1.1.42) are upregulated in *glu1-2 *leaves (Additional file 5). Although this could indicate an increase in cytosolic 2-oxoglutarate synthesis, it should however be noted that a role of the latter in cytosolic 2-oxoglutarate production has not yet been established.

#### Uptake, transport and distribution of nitrate and ammonium

Fd-GOGAT1 is a key enzyme in the primary assimilation of nitrogen and knocking it down was expected to change the transcriptional level of genes involved in nitrogen uptake and transport (Table 2).

The NRT1 and NRT2 nitrate transporter families [50] were however only marginally affected. Of the 53 genes that encode putative NRT1 nitrate transporters in *A. thaliana*, five were induced in *glu1-2 *leaves. Of these, NRT1.1 (*At1g12110*) has been implicated in stomatal aperture and drought stress [51] and its NO3^- ^sensing role in root architecture was recently described [52]. NRT1.5 (*At1g32450*) and NRT 1.7 (*At1g69870*) are less well characterised but have been shown to transport nitrate in a heterologous system [50]. The latter of these genes is also slightly induced in *glu1-2 *roots. On the other hand, eight genes encoding putative NRT1 family members, such as the NRT1.3/NTP3 (*At3g21670*; [53]), are downregulated in *glu1-2 *leaves. Out of the seven NRT2-encoding genes, only *At5g14570 *(*NRT2.7*) was moderately induced in *glu1-2 *leaves (Table 2).

An essential role in nitrate uptake has also recently been shown for the NAR2-like protein [54,55]. The gene *NRT3.1/NAR2.1 *(*At5g50200*) encoding this protein is induced in *glu1-2 *leaves (Table 2). None of the genes implicated in nitrate uptake and transport mentioned above was affected in *glu1-2 *roots.

The ammonium transporter (AMT) proteins, which are encoded by six genes in *A. thaliana*, are likely responsible for high affinity ammonium transport in plant roots [56]. The gene *At2g38290 *encoding the ammonium transporter AMT2;1 was upregulated in *glu1-2 *leaves (Table 2), but its contribution to ammonium uptake *in planta *has been questioned recently [57].

The nitrate/proton antiporter AtCLCa mediates nitrate accumulation in vacuoles [58,59] and its encoding gene *At5g40890 *is down-regulated in *glu1-2 *leaves (Table 2). Several NRT1 gene-encoding proteins are claimed to be involved in nitrate distribution in different cellular compartments and tissues, but detailed evidence has not been provided yet [50].

Two vacuolar tonoplast intrinsic proteins (AtTIP2;1 and AtTIP2;3) have been proposed to be responsible for NH_3 _transport across the tonoplast membrane in *A. thaliana *[60]. *AtTIP2;1 *(At3g16240) is downregulated in *glu1-2 *leaves, whereas *AtTIP2;3 *(*At5g47450*) is downregulated in *glu1-2 *roots (Table 2).

### NMR analysis of *glu1-2 *mutant leaves and roots reveal differences in amino acid contents

Although the Fd-GOGAT deficient *gluS/gls *mutants did not seem to be impaired in primary nitrogen assimilation [9], it was later shown that *Fd-GOGAT1/GLU1 *indeed plays a role in primary nitrogen metabolism [5]. As described above, knocking down *Fd-GOGAT1 *in the *glu1-2 *mutant affected several genes involved in primary nitrogen assimilation but the effects at the transcriptional level were more moderate than expected. Metabolite analysis by NMR spectroscopy of *glu1-2 *mutant tissue was performed to assess metabolic changes in the mutant.

**Figure 9:**
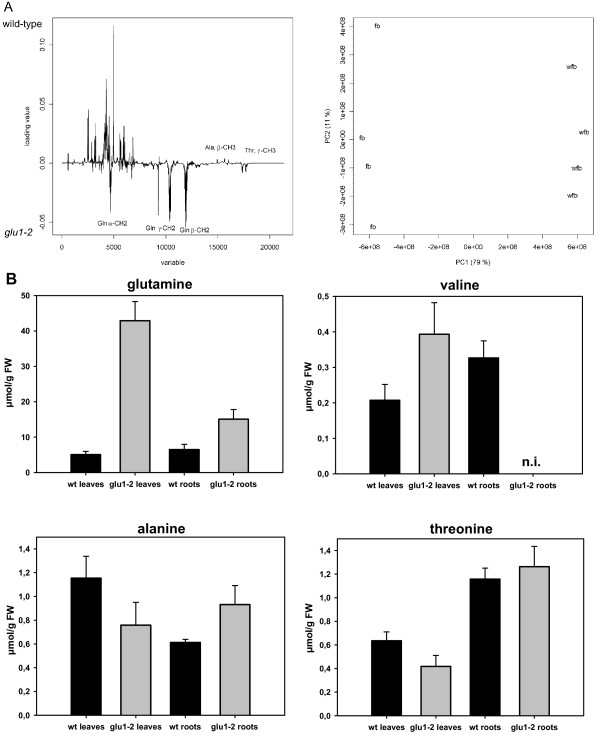
**Analysis of metabolic changes in the *glu1-2 *mutant by NMR spectroscopy**. Metabolic changes in leaves and roots of mutant (*glu1-2*) and wild-type (wt) plants were analysed by NMR spectroscopy. (**A**) The loading plot (left) for PC1 from the PCA analysis of leaves show the resonances that are different between the wild type and mutant groups (n = 4). Scatter plot (right) of *glu1-2 *mutant (fb) and wild-type (wfb) leaf samples. (**B**) The contents of glutamine, valine, alanine and threonine are given in μmol/g fresh weight ± SD (n = 4). Values for valine in *glu1-2 *roots could not be integrated (n.i.) due to overlapping resonances.

Using this technique, the four amino acids alanine, threonine, valine and glutamine were identified and some changes in their levels in the *glu1-2 *mutant were revealed (Table 3; Figure 9). Most importantly glutamine contents were increased more than eight-fold in *glu1-2 *leaves and more than two-fold in *glu1-2 *roots. This indicates that the loss of Fd-GOGAT1 is not compensated by Fd-GOGAT2 or NADH-GOGAT activity in the *glu1-2 *mutant. The fact that the latter two enzymes are more active in roots than in leaves [7] is consistent with a smaller increase in glutamine levels in *glu1-2 *roots than in leaves. Higher glutamine contents have been previously described for Fd-GOGAT deficient plants of *A. thaliana*, barley and tobacco [9-11,61]. Principal component analysis (PCA) of the NMR spectra indicates that glutamine is to a large extent responsible of the changes detected between the wild-type and the *glu1-2 *mutant (Figure 9A, left panel). PC1 explains 79% of the total explained variance in leaf samples and allows to discriminate between *glu1-2 *and wild-type leaf samples (Figure 9A, right panel). Conversely to the increase in glutamine, the levels of glutamate are expected to be reduced in the *glu1-2 *mutant. Reduced levels of glutamate have been described for Fd-GOGAT deficient barley and *A. thaliana *plants [10,61,62]. The levels of valine were also considerably increased in *glu1-2 *leaves, consistent with previous reports on Fd-GOGAT deficient tobacco plants ([11]; Figure 9B). This is however difficult to explain by the transcriptional profiling of the *glu1-2 *mutant. Indeed, only two genes of the valine biosynthetic pathway are slightly upregulated (Figure 8; Table 2) and genes involved in the degradation of valine are not affected. In addition, the diversion of this pathway towards leucine biosynthesis via the use of α-ketoisovalerate seems rather upregulated than downregulated (data not shown). Alanine content was reduced in *glu1-2 *leaves and increased in *glu1-2 *roots, although the changes were more moderate (Figure 9B). This could be related to the decrease in glutamate levels and increased reverse alanine:2-oxoglutarate aminotransferase activity, i.e. the conversion of alanine to pyruvate (Figure 8). Reduced foliar levels of alanine have previously been observed in Fd-GOGAT deficient barley and tobacco plants [11,61]. Threonine levels were only affected in *glu1-2 *leaves, showing a slight reduction (Figure 9B). In Fd-GOGAT deficient tobacco plants the levels of foliar alanine and threonine were respectively reduced and unaffected [11]. The reduced levels of alanine and threonine in *glu1-2 *leaves are accompanied by an overall upregulation in gene expression in the biosynthetic pathways of these amino acids (Figure 8). Other amino acids were not identified by NMR, but changes in several amino acids have been previously reported in Fd-GOGAT deficient plants [10,11].

**Table 3:** Contents of some amino acids in wild type and *glu1-2 *plants

		glutamine	valine	alanine	threonine
**Leaves**	**wild type**	5.07 ± 0.91	0.21 ± 0.04	1.15 ± 0.18	0.64 ± 0.07

	***glu1-2 *mutant**	42.90 ± 5.42	0.39 ± 0.09	0.76 ± 0.19	0.42 ± 0.09

**roots**	**wild type**	6.48 ± 1.51	0.33 ± 0.05	0.61 ± 0.03	1.16 ± 0.09

	***glu1-2 *mutant**	15.08 ± 2.71	n.i.	0.93 ± 0.16	1.26 ± 0.17

Glutamine, valine, alanine and threonine contents in *glu1-2 *and wild type leaves and roots measured by NMR spectroscopy. Values are expressed in μmol.g^-1 ^fresh weight ± standard deviation (n = 4). n.i.= not integrated due to overlapping resonances

Analysis with PathExpress identified starch and sucrose metabolism as being downregulated in leaves and roots of the *glu1-2 *mutant (see above). This is not surprising considering the close interaction between C and N metabolism through the GS/GOGAT cycle. Differences in the contents of sucrose and glucose were not observed by NMR spectroscopy of *glu1-2 *leaves and roots (data not shown). This is consistent with results from tobacco plants with reduced Fd-GOGAT activity [42].

### Secondary responses of the *glu1-2 *mutant revealed by transcriptional profiling

#### Photosynthesis and related biochemical processes are affected in *glu1-2 *mutant leaves

**Figure 10:**
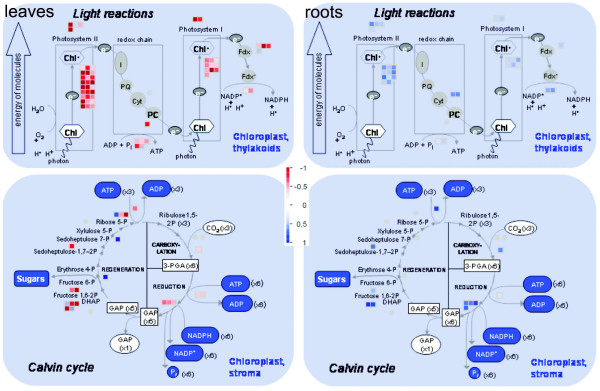
**Changes in gene expression in photosynthesis-related pathways of the *glu1-2 *mutant**. Expression changes of genes involved in photosynthesis in *glu1-2 *mutant leaves (**left**) and roots (**right**) using the MapMan software. Represented are only the genes showing a significant (P = 0.01) change in expression between the *glu1-2 *mutant and the wild-type and that have been attributed to the respective bins by MapMan. Genes whose expression is increased or decreased in the *glu1-2 *mutant tissue versus the corresponding wild-type tissue are shown by an increasingly intense blue and red colour, respectively. The graduation can be seen on the scale that is represented in the centre of the figure.

Photosynthesis and related biochemical processes were recurrently identified in our unbiased search of affected pathways (Additional files 2, 3 and 4). Especially the GO term GO:0015979 (photosynthesis), and to a lesser extent GO:0019684, GO:0042548 and GO:0042549, was revealed by GOstat as being affected in several of our datasets (Additional file 2). If only the GO terms related to photosynthesis that were identified by GOstat as being affected are taken into consideration, 37 genes show different expression levels in *glu1-2 *leaves (Additional file 6). All but one of these are repressed and the level of repression varies from log_2 _= -0.32 to -1.76. Although the TAIR database to some extent categorises different genes into these GO terms, these are also almost exclusively downregulated (Additional file 6). If all genes included in GO:0015979 and its subcategories as provided by AmiGO are taken into condiseration, the list contains 49 unique genes not identified by GOstat whose expression is affected in the *glu1-2 *mutant leaves. Of these only five genes are (moderately) upregulated. The photosynthesis related bins of the MapMan software (Figures 4 and 10) identify another 71 genes with changed expression in *glu1-2 *leaves. All but ten of these are downregulated (Additional file 6). Hence, a total of more than 150 genes attributed to photosynthesis related pathways are affected in *glu1-2 *leaves, of which approximately 90% are downregulated. In *glu1-2 *roots, 52 genes attributed to the photosynthesis related GO terms or bins show a differential expression and these are exclusively induced (Additional file 6).

Several genes involved in the Calvin cycle are thus affected in the *glu1-2 *mutant, with most of them being downregulated in leaves and upregulated in roots (Figure 10). The most downregulated gene in the Calvin cycle in *glu1-2 *leaves is encoding a putative fructose-biphosphate aldolase (*At4g26530*).

**Figure 11:**
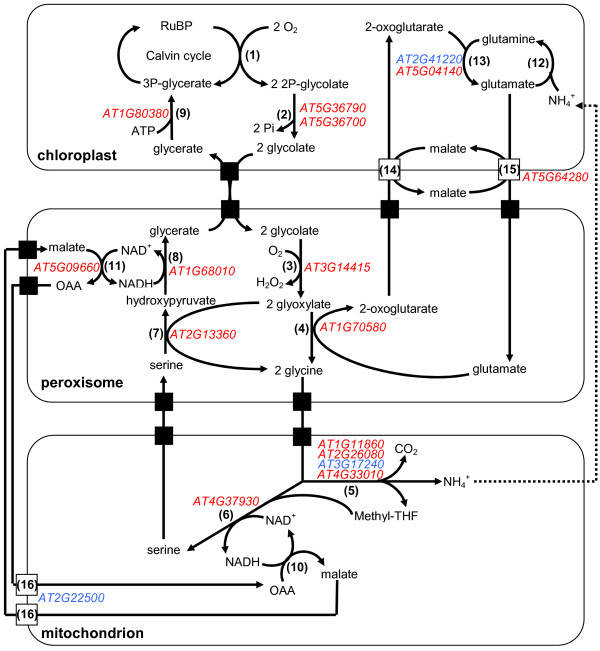
**Changes in gene expression in the photorespiratory cycle of the *glu1-2 *mutant leaves**. Schematic representation of the photorespiratory cycle and indication of the genes that show different expression levels in *glu1-2 *leaves compared to wild-type leaves. Gene IDs indicated in blue are upregulated whereas those indicated in red are downregulated in the *glu1-2 *mutant. Genes involved in the Calvin cycle have been omitted from this figure (see Figure 10 for this purpose). Enzymes: (1) ribulose-1,5-biphosphate carboxylase/oxygenase, (2) phosphoglycolate phosphatase, (3) glycolate oxidase, (4) glutamate:glyoxylate aminotransferase, (5) glycine decarboxylase complex, (6) serine hydroxymethyltransferase, (7) serine:glyoxylate aminotransferase, (8) hydroxypyruvate reductase, (9) glycerate kinase, (10) mitochondrial malate dehydrogenase, (11) peroxisomal malate dehydrogenase, (12) glutamine synthetase 2, (14 and 15) chloroplast dicarboxylate transporter 1 and 2, (16) mitochondrial dicarboxylate carriers. Black boxes indicate putative transporters. The detailed data of the genes depicted in this figure and the affected genes in *glu1-2 *roots are presented in Table 4. Abbreviations used: OAA: oxaloacetate; RuBP: ribulose-1,5-biphosphate; THF: tetradydrofolate.

A process directly linked to photosynthesis and the Calvin cycle is photorespiration, a consequence of the oxygenation of ribulose-1,5-biphosphate (RuBP) by RuBP carboxylase/oxygenase [3]. This oxygenation produces 3-phosphoglycerate and 2-phosphoglycolate, the latter being recycled into 3-phosphoglycerate by the photorespiratory cycle. In this process O_2 _is consumed in the plastid and CO_2 _and ammonium are released in the mitochondria. The released ammonium is reassimilated by the GS/GOGAT pathway in the plastid (for review: [63]). The essential role that glutamate synthase plays in this process is evidenced by the fact that mutants deficient in Fd-GOGAT activity exhibit a photorespiratory-dependent lethal phenotype [5,9]. Transcriptional profiling of the *glu1-2 *mutant indeed shows that genes involved in most of the steps of the photorespiratory cycle are downregulated in leaves (Figure 11; Table 4). These include the genes encoding phosphoglycolate phosphatase (*PGLP1*; *At5g36700*) and glycerate kinase (*GLYK*; *At1g80380*) of plastids and mitochondrial serine hydroxymethyltransferase (*SHM1*; *At4g37930*). Knock-out mutants of these genes exhibit a conditional lethal photorespiratory phenotype [31,64,65]. The gene *At1g68010 *encoding the peroxisomal NADH-dependent hydroxypyruvate reductase HPR1 is downregulated in *glu1-2 *leaves with no concomitant change of *At1g79870 *encoding the cytosolic isoform HPR2 that provides a cytosolic bypass to the photorespiratory cycle [66]. The gene encoding the dicarboxylate transporter DiT2.2 is also repressed in *glu1-2 *leaves. Its role is yet unclear, although the homolog DiT2.1 has been identified as a glutamate/malate translocator with an essential role in photorespiration [67]. A notable exception to the general downregulation of photorespiration-related genes is *At2g22500 *encoding one of the three recently characterised mitochondrial dicarboxylate carriers (DIC1-3) that are able to transport malate and oxaloacetate among other substrates [68]. In *glu1-2 *roots the expression of a smaller number of genes involved in photorespiration is affected by the *glu1-2 *mutation, and all these are upregulated (Table 4). It should also be noted that the changes in expression levels for both leaves and roots are moderate, which may explain the fact that our *glu1-2 *mutant has a less severe photorespiratory phenotype than the Fd-GOGAT1 mutants characterised previously [5,9]. In addition, Takahashi et al. [69] showed that mutants impaired in photorespiration have accelerated photoinhibition of the photosystem II and that this is due to a suppressed repair process through inhibition of the D1 protein translation.

**Table 4:** Genes that are involved in the photorespiratory pathway and that are differentially expressed between the *glu1-2 *mutant and the wild-type mutant

EC #	Enzyme/protein	Abbreviation	Gene ID	log_2 _root	log_2 _leaf
3.1.3.18	phosphoglycolate phosphatase	putative PGLP AtPGLP1	*AT5G36790* *AT5G36700*	0.674	-0.658

1.1.3.15	glycolate oxidase	GOX	*AT3G14415* *AT3G14420*	0.655	-0.464

2.6.1.2/2.6.1.4	glutamate:glyoxylate aminotransferase	GGAT2	*AT1G70580*		-0.680

	glycine decarboxylase complex -- H protein	AtGDH3	*AT1G32470*	0.593	

	glycine decarboxylase complex -- H protein	AtGDH1	*AT2G35370*	0.768	

1.8.1.4	glycine decarboxylase complex -- L protein	AtmLPD1	*AT3G17240*		0.689

1.4.4.2	glycine decarboxylase complex -- P protein	AtGLDP1	*AT4G33010*	0.439	-1.126

1.4.4.2	glycine decarboxylase complex -- P protein	AtGLDP2	*AT2G26080*		-0.532

2.1.2.10	glycine decarboxylase complex -- T protein	AtGDT1	*AT1G11860*		-0.600

2.1.2.1	serine hydroxymethyltransferase	SHM1	*AT4G37930*		-0.513

2.6.1.45	serine:glyoxylate aminotransferase	AtSGT	*AT2G13360*		-0.566

1.1.1.29	peroxisomal hydroxypyruvate reductase	HPR1	*AT1G68010*	0.705	-0.689

2.7.1.31	glycerate kinase	GLYK	*AT1G80380*		-0.554

1.1.1.37	glyoxysomal malate dehydrogenase	MDHG1/ pMDH2	*AT5G09660*		-0.851

1.4.1.7	Fd-dependent glutamate synthase	Fd-GOGAT 1/GLU1	*AT5G04140*		-5.738

1.4.1.7	Fd-dependent glutamate synthase	Fd-GOGAT 2/GLU2	*AT2G41220*		1.070

	chloroplast dicarboxylate transporter	DiT2.2	*AT5G64280*		-0.439

	mitochondrial dicarboxylate transporter	DIC1	*AT2G22500*		0.702

List of genes encoding enzymes involved in the photorespiratory pathway and whose expression is affected by knocking down *Fd-GOGAT1 *in the *glu1-2 *mutant. See also Figure 11. The presence of two gene IDs in some boxes indicates a nondiscriminating probe, i.e. the change in expression can not be attributed to a specific gene.

Due to the dual-targeting of glutamine synthetase 2 (GS2) to the plastid and mitochondria [22], other schemes of the reassimilation of ammonium than the photorespiration cycle depicted (Figure 11) and discussed here, have been proposed [2].

**Figure 12:**
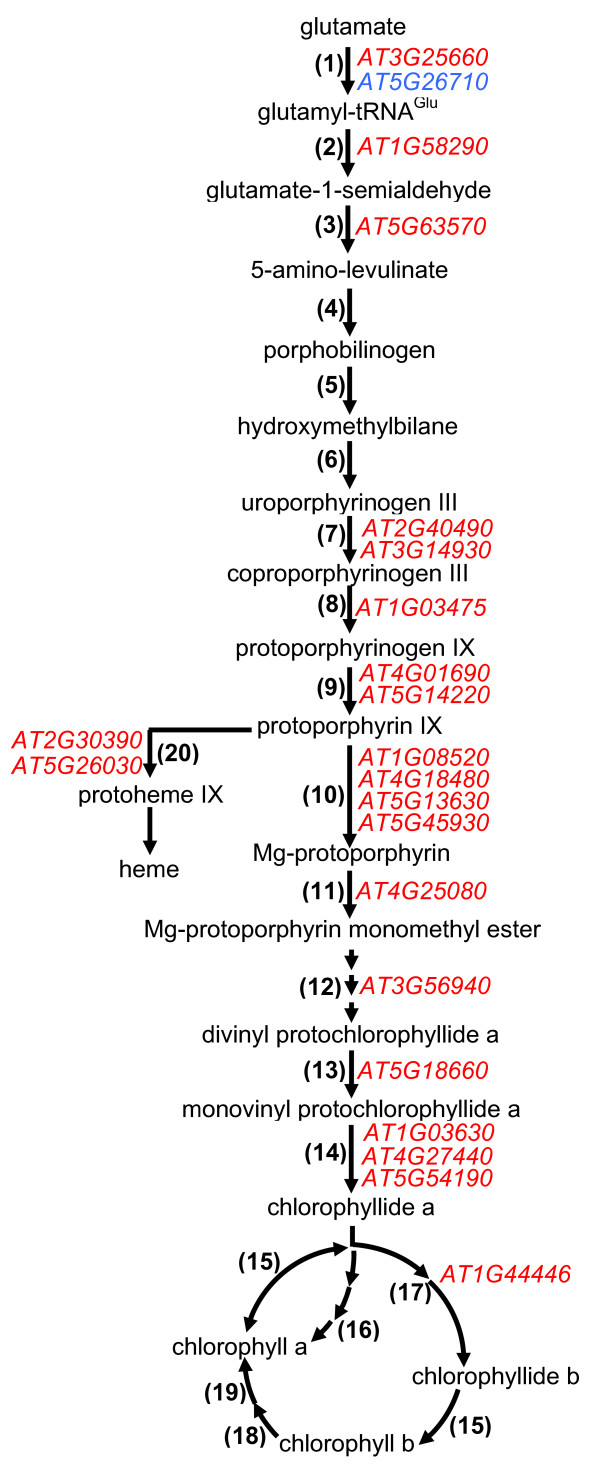
**Changes in gene expression in chlorophyll biosynthesis of the *glu1-2 *mutant leaves**. Schematic representation of chlorophyll biosynthesis and the genes showing different expression levels in *glu1-2 *leaves. Gene IDs indicated in blue are upregulated whereas those indicated in red are downregulated. Enzymes: (1) glutamate-tRNA ligase, (2) glutamyl-tRNA reductase, (3) glutamate-1-semialdehyde 2,1-aminomutase, (4) porphobilinogen synthase, (5) hydroxymethylbilane synthase, (6) uroporphyrinogen-III synthase, (7) uroporphyrinogen decarboxylase, (8) coproporphyrinogen oxidase, (9) protoporphyrinogen oxidase, (10) magnesium chelatase, (11) magnesium protoporphyrin IX methyltransferase, (12) magnesium protoporphyrin IX monomethyl ester cyclase, (13) 3,8-divinyl protochlorophyllide 8-vinyl reductase, (14) protochlorophyllide oxidoreductase, (15) chlorophyll synthase, (16) geranylgeranyl reductase, (17) chlorophyllide a oxygenase, (18) chlorophyllide b reductase, (19) 7-hydroxy chlorophyllide a reductase, (20) ferrochelatase. Reduced acceptors and cofactors are omitted from the figure. The detailed data of the genes depicted in this figure and the affected genes in *glu1-2 *roots are presented in Additional file 6.

Chlorophyll is synthesised from glutamate. Many of the reactions of the chlorophyll biosynthetic pathway are downregulated (Figure 12), although the fold changes in expression are moderate (Additional file 6). However, two genes (*At1g03630 *and *At5g54190*) encoding protochlorophyllide reductases that catalyse the last step leading to the synthesis of chlorophyllide *a *(Figure 12), show a more pronounced downregulation. Downregulation of genes implicated in chlorophyll biosynthesis and other photosynthesis related pathways, such as photorespiration (discussed above), in *glu1-2 *mutant leaves are consistent with its chlorotic phenotype (Figure 2). A chlorotic phenotype has been observed and reduced total chlorophyll contents have been measured in Fd-GOGAT deficient plants of *A. thaliana*, tobacco and barley [5,9,11,61]. Barley plants with reduced Fd-GOGAT activity have also been shown to have a reduced chlorophyll *a/b *ratio [61]. Takahashi et al. [69] hypothesised that depletion of glutamate in the Fd-GOGAT mutant may lead to accumulation of glyoxylate that may be involved in a decline of the photosynthetic rate.

#### The *glu1-2 *mutant displays a multiple stress response

Categorisation of genes affected in leaves and roots of the *glu1-2 *mutant presented above showed a high number of genes related to stimulus and stress responses. More detailed analysis of the affected genes revealed that these changes in expression can not be attributed to one particular stress. Indeed, genes responsive to a multitude of different abiotic stresses, including light, drought, salt, heat and cold, oxidative stress and osmotic stress were affected (Additional file 2). This indicates that knocking down *Fd-GOGAT1 *leads to a secondary response that consists in the activation of multiple stress responses or the activation of mechanisms that are common to several stresses. Due to the extensive nature of these transcriptional responses, only a selection will be presented briefly in the following section and the reader is kindly referred to Additional files 7 to 14 for the details on the affected genes.

##### Key stress responsive transcription factors affected in the *glu1-2 *mutant

Numerous genes (putatively) encoding transcription factors belonging to different families, such as the zinc finger proteins (WRKY, C2H2, CCCH), MYBs and bHLHs (Additional file 7), were affected in the *glu1-2 *mutant.

WRKY25, whose encoding gene *At2g30250 *showed the highest induction of WRKY transcription factors in *glu1-2 *leaves, has been implicated in the defence against *Pseudomonas syringae *[70]. *WRKY25 *expression has also been shown to be induced in response to heat shock, wounding and oxidate stress. Higher levels of *WRKY25 *transcripts were detected in cytosolic ascorbate peroxidase Apx1-deficient plants, which maintain a high steady state level of H_2_O_2 _and activate ROS defence mechanisms [71].

The C2H2-type zinc finger transcription factor ZAT12 plays an important role in oxidative and abiotic stress response [71-73] and its encoding gene *At5g59820*, is upregulated in *glu1-2 *mutant leaves. *ZAT12 *expression is, like *WRKY25*, induced by heat shock, wounding and oxidate stress and higher transcripts were also detected in cytosolic ascorbate peroxidase (Apx1)-deficient plants [71]. ZAT12 expressing plants can tolerate oxidate stress [71] and show an increased freezing tolerance [73].

The genes encoding the two MYB-related transcription factors LHY (*At1g01060*) and CCA1 (*At2g46830*), which are associated with the circadian clock and act as negative regulators of the periodic flowering pathway [74], were strongly downregulated in *glu1-2 *leaves respectively. A role of LHY and CCA1 in the response to abiotic stresses has also been proposed [75].

Five genes (putatively) encoding bHLH transcription factors were among the 5% most repressed genes in *glu1-2 *leaves. While a role for *BHLH101 *(*At5g04150*) has not been described yet, it is phylogenetically closely linked to *BHLH039 *(*At3g56980*; [76]) which is also strongly repressed in *glu1-2 *leaves. *BHLH039 *has been shown to be downregulated by jasmonic acid (JA), and induced by salicylic acid (SA) [77] and iron deficiency [78]. It should be noted that these two transcription factors are also downregulated in *glu1-2 *roots.

*DREB2C/DREB2H *(dual probe *At2g40340*/*At2g40350*) are highly upregulated AP2/ERF transcription factors in *glu1-2 *leaves. *DREB2C *has been described as being heat and drought inducible and overexpression confers thermotolerance to the plants. Analysis of these plants lead the authors to hypothesise that DREB2C is a late regulator of genes under heat stress [79].

##### Flavonoid biosynthesis is affected in the *glu1-2 *mutant

Flavonoids, which were revealed as affected in the gene sets, are a class of compounds that act for example as protection against abiotic and biotic stresses and their concentrations increase in response to these [80].

**Figure 13:**
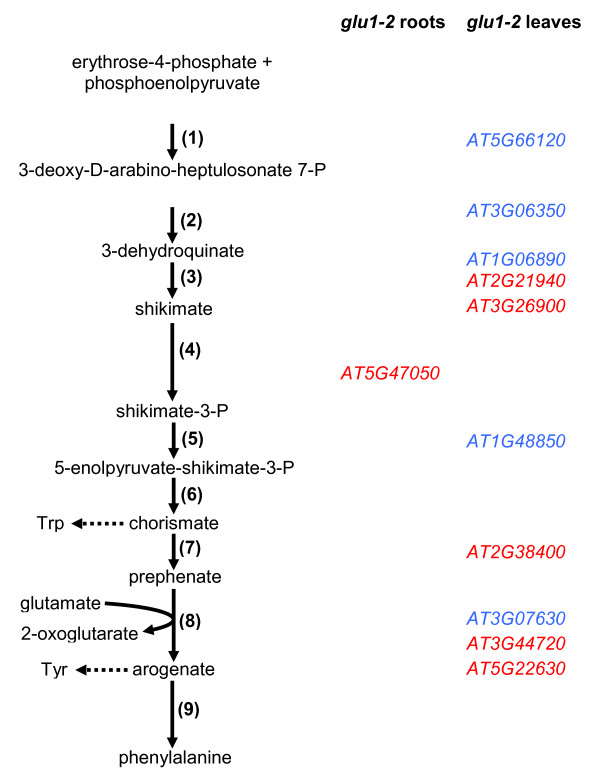
**Changes in gene expression in the shikimate pathway of the *glu1-2 *mutant**. Simplified schematic representation of the shikimate pathway and the genes showing different expression levels in *glu1-2 *leaves and root. Gene IDs indicated in blue are upregulated whereas those indicated in red are downregulated. Enzymes: (1) 3-deoxy-D-D-arabino-heptulosonate 7-phosphate synthase (DAHPS), (2) 3-dehydroquinate synthase (DQS), (3) 3-dehydroquinate dehydratase/shikimate dehydrogenase (DHQD), (4) shikimate kinase, (5) 5-enolpyruvylshikimate-3-phosphate synthase, (6) chorismate synthase, (7) chorismate mutase, (8) prephenate aminotransferase, (9) arogenate dehydratase. The figure is based on [80]. The detailed data of the genes depicted in this figure are presented in Additional file 8.

**Figure 14:**
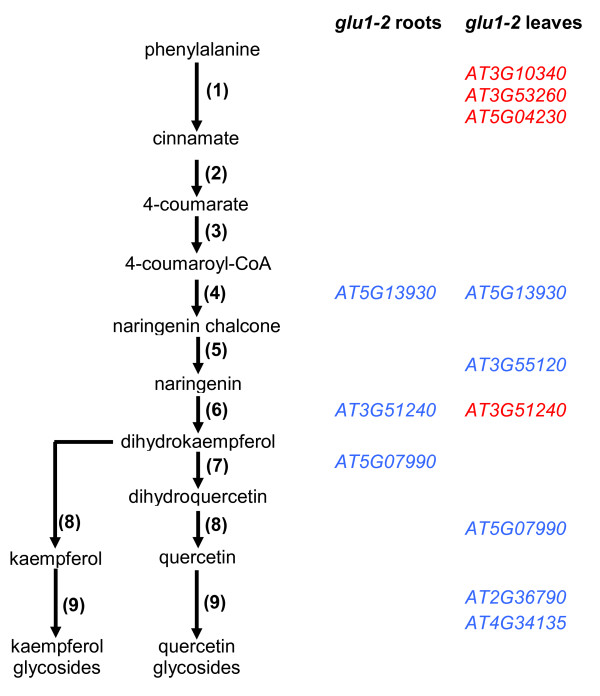
**Changes in gene expression in flavonoid biosynthesis of the *glu1-2 *mutant**. Simplified schematic representation of the flavonoid biosynthetic pathway and the genes showing different expression levels in *glu1-2 *leaves and root. Gene IDs indicated in blue are upregulated whereas those indicated in red are downregulated. Enzymes: (1) phenylalanineammonia lyase (PAL), (2) cinnamate 4-hydroxylase, (3) 4-coumarate-CoA ligase, (4) chalcone synthase, (5) chalcone isomerase, (6) flavanone 3-hydroxylase, (7) flavonoid 3'-hydroxylase, (8) flavonol synthase, (9) flavonol glycosyltransferase. The figure is based on [80]. The detailed data of the genes depicted in this figure are presented in Additional file 8.

Flavonoids are produced from phenylalanine which is synthesised through the shikimate pathway (Figures 13 and 14). In leaves of the *glu1-2 *mutant the phenylalanine metabolism was identified as being negatively affected whereas flavonoid biosynthesis was identified as being positively affected on the transcriptional level (Additional file 2). This could be interpreted as reduced levels of phenylalanine and increased amount of flavonoids and related compounds in *glu1-2 *mutant leaves, although tobacco plants with reduced Fd-GOGAT activity accumulate phenylalanine and tyrosine [11].

Although the expression levels of a fair number of genes implicated in the shikimate pathway were affected in *glu1-2 *leaves (Figure 13), a more detailed analysis shows that most genes are only moderately affected. In addition, no clear trend towards up- or downregulation of the pathway is visible (Additional file 8). Indeed, on one hand the genes encoding 3-deoxy-D-arabino-heptulosonate 7-phosphate synthase DAHPS that have been identified as important targets for regulation of the shikimate pathway are not affected in the *glu1-2 *mutant. On the other hand, the genes encoding 3-dehydroquinate synthase (DQS) and 3-dehydroquinate dehydratase (DHQD) are slightly induced in *glu1-2 *leaves but do not constitute important regulatory steps [80]. One of two putative prephenate aminotransferase-encoding genes (*At2g38400*) is downregulated in *glu1-2 *leaves. Prephenate aminotransferase transfers the amino group from glutamate or aspartate for the synthesis of arogenate. Genes putatively encoding arogenate dehydratase that catalyses the last step of the shikimate pathway leading to the formation of phenylalanine, were either induced, repressed or unaffected in *glu1-2 *leaves (Figure 13). This complex behaviour of the shikimate pathway in response to various treatments and its regulation by feedback mechanisms has been described [80].

As to the flavonoid pathway as such (Figure 14), it may be worth pointing out that although three (PAL2 to 4) of the four genes encoding phenylalanine ammonia lyases that putatively catalyse the first step of the phenylpropanoid pathway [80] are slightly downregulated in *glu1-2 *leaves, PAL1, whose effect on flavonoid accumulation has been shown, was not affected. Among the various affected genes was the gene *At5g13930 *encoding chalcone synthase, the first committed enzyme in flavonoid synthesis, which was upregulated in leaves and roots of the *glu1-2 *mutant. *CHS *expression has been shown to be induced by sugar, high light, UV and blue light, and phoshorus and nitrogen depletion [80]. Two genes encoding UGTs that catalyse *in vitro *the transfer of glucose from UDP-glucose to the 7-OH position of flavonols were among the twenty most highly upregulated ones in *glu1-2 *leaves: UGT73B1 [81] and UGT73C6 [82]. UGTs that are involved in flavonoid biosynthesis are essential for the accumulation of flavonoids [80]. It should however be noted that the UGT73C6 is recognised by a probe that also recognises UGT73C5 and the increased signal could therefore also be due to an increased expression of the latter. UGT73C5 is responsible for glycosylating brassinosteroids [83].

In *glu1-2 *roots, PathExpress identifies flavonoid biosynthesis in both down- and upregulated genes, whereas it was revealed by GeneBins only among downregulated genes (Additional files 3 and 4). This downregulation of genes in roots is however hard to sustain by looking at the reduced number of affected genes whose role in this pathway has been confirmed. As to the regulators of the flavonoid biosynthetic pathway, PAP1/MYB75 is the only implicated transcription factor that is affected in *glu1-2 *roots, where it is slightly upregulated (Additional file 8). PAP1/MYB75 is a positive regulator of the flavonoid biosynthetic pathway and is strongly induced by nitrogen deficiency [80]. However the genes encoding PAP2 and MYB12, two other transcription factors of this pathway that are also induced by nitrogen deficiency [80], are not affected in the *glu1-2 *mutant.

The multidrug resistance-related protein (MRP)-type ABC transporters have been implicated in the vacuolar sequestration of phenolic compounds such as flavonoids and anthocyanins, and flavonoids have been suggested as negative regulators of MDR-type members (for review: [84]). A long distance transport of flavonoids by the MRP-type of ABC transporters was also recently suggested, although the transporter and the mechanism were not identified [85]. In total, 11 of the 15 AtMRP-encoding genes are induced in *glu1-2 *leaves, whereas only two are affected in *glu1-2 *roots (Additional file 9). The AtMRP2 to AtMRP5 are ATP-dependent pumps for organic ions [86] and, interestingly, AtMRP2 and AtMRP3 are able to transport glutathione S-conjugates and chlorophyll catabolites into vacuoles [87,88]. AtMRP4 [89] and AtMRP5 are supposedly involved in regulating ion channel activities in guard cells but also have transport activity: folate for AtMRP4 and E_2_17G for AtMRP5. Folate mediates large metabolic fluxes mainly during photorespiration as a cofactor of the mitochondrial GDC/SHM (mentioned before) complex [86]. The roles of the other MRP transporters affected in *glu1-2 *leaves have not yet been described, although the contribution of AtMRP12 in detoxification seems to be marginal [88] and AtMRP15 may possibly constitute a pseudogene [90]. Based on gene expression, Klein et al. [86] speculate that AtMRP14 could be involved in processes controlling seed integrity and germination efficiency such as the regulation of dormancy.

##### Cytochrome P450 monooxygenase-encoding genes are affected in the *glu1-2 *mutant

Another group of enzymes related to stress response are cytochrome P450 monooxygenases. They are known to be involved in the synthesis of structural components, hormones, signalling molecules (e.g. SA and JA) and defense compounds (flavonoids, phytoalexins, glucosinolates). A large number of cytochrome P450s are responsive to hormones, signaling molecules and environmental stresses (for review: [91]). The functions of most of the 245 members of this group of enzymes in *A. thaliana *have not yet been identified, and it is therefore impossible to attribute most of these to particular pathways. Cytochrome P450s are categorised into the gene ontology term GO:0006118 (electron transport), which was revealed as being overrepresented (together with its parent GO:0006091) by GOstat analysis (Additional file 2).

In the *glu1-2 *mutant cytochrome P450 were especially frequent among the most upregulated and downregulated genes in roots. In total 22 and 39 genes annotated as encoding cytochrome P450 are affected in roots and leaves respectively (Additional file 10), indicating that proportionally a greater number of cytochrome P450s were responsive in roots.

Of the 22 genes affected in roots, 9 are repressed and 13 are induced (Additional file 10). Five cytochrome P450 encoding genes (*CYP712A1*, *76G1*, *93D1*, *716A2*, *96A12 *in descending order of induction) are among the 10% most highly induced genes and all but one are specifically affected in roots. On the other hand, the genes encoding the cytochrome P450s CYP71A12, CYP82C4 and CYP81F2 were among the 10% most highly repressed genes in *glu1-2 *roots. The function of CYP71A12and CYP82C4 has not yet been described. It was however shown that *CYP71A12 *expression responded in a similar way to aphid infestation than *CYP71B15*, which is involved in synthesis of the defence compound camalexin [92]. The role of CYP81F2 in the synthesis of indole glucosinolates, another group of compounds involved in plant defence, was recently reported [93]. In the *glu1-2 *mutant these three genes are repressed in roots while they are induced in leaves. The genes encoding three cytochrome P450 (i.e. CYP79B2, CYP79B3 and CYP83A1) involved in the biosynthesis of glucosinolates are on the other hand induced in *glu1-2 *roots.

In *glu1-2 *leaves, 20 cytochrome P450s were induced whereas 16 were repressed (Additional file 10). The two cytochrome P450-encoding genes that show the highest induction in *glu1-2 *leaves are *At3g28740 *(*CYP81D11*) and *At4g37370 *(*CYP81D8*). These genes, together with *At3g14660 *(*CYP72A13*), were among the 30 genes upregulated by cis-jasmone treatment [94]. *CYP81D8 *is also among the eight genes induced early on by imidazolinone treatment, a herbicide inhibiting acetohydroxyacid synthase (AHAS; EC 2.2.1.6) catalysing the first step of valine, isoleucine and leucine synthesis (Figure 8; [95]). Expression of *CYP81D11 *is induced by the application of various chemicals, such as 2,4-D and SA, and bacterial infestation [96]. These and other cytochrome P450-encoding genes upregulated in *glu1-2 *leaves are also particularly responsive to abiotic stresses such as oxidative stress, osmotic stress, cold, heat and salt [97]. Analysis of genes co-expressed with *CYP81D8 *and *CYP81D11 *[97] could also indicate a role in phenylpropanoid biosynthesis. Similarly, cytochrome P450 genes showing the highest downregulation in leaves could be associated with photosynthesis related processes, based on co-expression analysis [97].

##### The detoxification of secondary metabolites and xenobiotics is induced in *glu1-2*

Another indication that *glu1-2 *mutants deploy a general stress response is the change in expression of genes involved in the detoxification of secondary metabolites and xenobiotics.

Some of the cytochrome P450-encoding genes that are affected in the *glu1-2 *mutant may play a role in the phase I of this detoxification [98].

Glutathione S-transferases (EC 2.5.1.18; GSTs), also known as glutathione transferases (GTs), are involved in the second phase of detoxification processes. These enzymes catalyse the conjunction of glutathione (GSH-tripeptide) with electrophilic compounds, to form non toxic derivatives that are ready to be compartimentalised in vacuoles. In addition, GSTs can serve as peroxidases, isomerases and thiol transferases or have non-catalytic functions such as ligand binding and modulation of signalling processes (for review: [99]). Several studies that have analysed the responsiveness of AtGSTs to different stimuli have revealed the complex nature of the regulation of these genes (for review: [100]). In *glu1-2 *leaves the expression of 26 genes coding for (putative) GSTs belonging to the tau (GSTU), phi (GSTF) and zeta (GSTZ) classes are affected, 20 of these being induced and 6 being repressed. In *glu1-2 *roots, four glutathione S-transferases are induced and two are repressed (Additional file 11). Eight GSTU-encoding genes are among the 5% most upregulated genes in *glu1-2 *leaves and *AtGSTU24 *(*At1g17170*), the most highly induced of them, was reported to be induced by herbicide treatment, xenobiotic exposure and in the catalase 2 deficient mutant (characterised by intracellular redox perturbation and activation of oxidative signalling). It was hypothesised that AtGSTU24 could be involved in the conjugation of stress-induced catabolites ([101] and references therein). *AtGSTU24 *has also been shown to be SA inducible [102]. Several of the other highly upregulated GSTU-encoding genes, such as GSTU1, 2, 4, 7, 9, 19, 22, 25 are induced in response to salt stress [103]. As to members of the phi class that are induced in *glu1-2 *leaves, *AtGSTF6/GSTF7 *(*At1g02930*/*At1g02920 *dual probe) are responsive to cold and heat stress, oxidative damage and metal exposure [104]. *AtGSTF2 *has also been shown to be responsive to several stimuli and the protein interacts with flavonoids *in vitro *[105].

UDP-glycosyltransferases (UGTs) use UDP-activated sugars as donor to catalyse the glycosylation of various metabolites and are hence implicated in a series of mechanisms and pathways, including phase II of the detoxification mechanism.

In *glu1-2 *mutant leaves 45 UGT-encoding genes are affected, which represents a third of the approximately 120 UGT-encoding genes that have been identified in the *A. thaliana *genome (for review: [106]). Thirty-six are induced in *glu1-2 *leaves and fourteen of these are among the top 5% induced genes (Additional file 12). UGT73B1 and UGT73C6, already mentioned above, are involved in flavonoid biosynthesis [81,82]. In vitro essays with UGT73B4 (*At2g15490*) showed that it was able to glycosylate both the 3-OH and the 4-OH position of the benzoate derivative 3,4-dihydroxybenzoic acid [107]. UGT75B1 (*At1g05560*) is associated with the callose synthase complex [108] and is highly induced in oxidative stress catalase 2 deficient mutants [101]. *UGT84A3 *(*At4g15490*) may be involved in sinapate ester metabolism in plants [109]. Three genes encoding UGTs that may be involved in cytokinin glycosylation [110] were also affected in *glu1-2 *leaves: *UGT73C1 *(*At2g36750*), *UGT85A1 *(*At1g22400*) and *UGT76C1 *(*At5g05870*). Recombinant UGT73C1 is also able to conjugate transformation products of the explosive 2,4,6-trinitrotoluene [111]. UGT84B1 (also known as IAGLU) glucosylates indole-3-acetic acid [112] and the encoding gene *At4g15550 *is also upregulated in *glu1-2 *leaves. UGT74F2 (*At2g34820*) has been described as a salicylic acid glucosyltransferase [107,113] and it has also been hypothesised as playing a role in tryptophan biosynthesis [114].

Several of the UGT-encoding genes that are highly upregulated in *glu1-2 *leaves have been shown to be stress responsive. Recently, *UGT74E2 *(*At1g05680*) was identified as one of eight genes that were rapidly and highly induced upon treatment with the herbicide imidazolinone [95]. This gene was the most highly induced in *glu1-2 *leaves. UGT73B1, UGT73B2 and UGT73B3 have been implicated in the response to oxidative stress and a role in stress response and resistance to *Pseudomonas syringae *was shown for UGT73B3 and UGT73B5 [115,116].

Phase III of xenobiotic detoxification in plants consists of storage of the compounds produced by the mechanisms of two first phases. The multidrug and toxic compound extrusion (MATE) efflux carriers may function in this process, although the transport activities and exact roles of most of these have not yet been described [117]. Fourteen of the 58 MATE protein-encoding gene of *A. thaliana *are induced and five are repressed in *glu1-2 *leaves (Additional file 13). Three are among the top fourteen induced genes in *glu1-2 *leaves: *AtDTX1 *(*At2g04040*), *AtDTX3 *(*At2g04050*) and *AtDTX4 *(*At2g04070*). AtDTX1 serves as an efflux carrier for plant derived alkaloids [118]. To our knowledge, the functions of AtDTX3 and AtDTX4 have not yet been reported but AtDTX4 was also among the genes that were rapidly induced by the herbicide imidazolinone [95].

##### Numerous other responsive genes linked to a number of different stresses/stimuli are induced in *glu1-2 *leaves

Besides the stress responsive genes and those involved in detoxification mechanisms that were discussed in more detail above, numerous other stress-related genes were affected in the *glu1-2 *mutant. It is beyond the scope of the present article to describe these in detail. This can however be exemplified by the striking overlap between the genes induced in leaves of the *glu1-2 *mutant and those described by Vanderauwera et al. [119] in the catalase 2 deficient CAT2HP1 plant. Of the 55 genes induced more than 3 fold in the CAT2HP1 plant, 45 were also induced in *glu1-2 *mutant leaves (Additional file 14), including cytochrome P450-, GST- and UGT-encoding genes already mentioned above. Other similarities in transcriptional responses can be detected between *glu1-2 *mutant plants and plants submitted to cold treatment [73], heat stress [120] and herbicide treatment [95](data not shown). This also exemplifies that the transcriptional response of the *glu1-2 *mutant bears the signatures of a multiple stress response.

## Conclusions

Knocking down the expression of the gene *Fd-GOGAT1 *coding for one of the two ferredoxin-dependent glutamate synthases, which catalyse the ultimate step in the biosynthesis of glutamate, and a key enzyme in the assimilation of inorganic nitrogen, has marked effects on the transcriptome of the plant as evidenced by our microarray analysis of the *glu1-2 *mutant. Even more so as the assayed plants were grown *in vitro *on 1x MS media supplemented with 3% sucrose, and nitrogen supply should not be limiting either under these conditions. Hence, the transcriptional profiling should be viewed under the angle that the growth conditions that were used in the present study do not constitute the most severe conditions possible for the *glu1-2 *mutant. This is also indicated by the fact that the *glu1-2 *mutant described here develops more slowly than wild-type plants when grown on soil, but does eventually complete its life cycle (data not shown). The Fd-GOGAT1 deficient *A. thaliana *mutants described previously display a lethal phenotype [5,7,9]. It should however be noted that the growth conditions for the *glu1-2 *mutant used here, did not prevent plants from showing a chlorotic phenotype, although less severe than in previously published studies.

As *Fd-GOGAT1 *expression is much higher in leaves than in roots of *A. thaliana *at the steady state ([5]; Additional file 1), the effects were expectedly of a larger scale in the leaves than in the roots, both in regard to the number of affected genes and the levels of regulation. The level of downregulation of *Fd-GOGAT1 *itself in the *glu1-2 *mutant is also much more pronounced in leaves than in roots.

Although an effect can be seen on the expression levels of genes involved in primary nitrogen assimilation, glutamate metabolism and related pathways, the number of such genes affected and the scale of changes are more moderate than we expected. This may be explained by the fact that the defect in primary nitrogen assimilation exhibited by Fd-GOGAT mutants are specific to conditions when photorespiration is suppressed [5], which is not the case under the experimental conditions chosen here. Upregulation of *Fd-GOGAT 2 *and *NADH-GOGAT *may also compensate to some extent for the loss of *Fd-GOGAT1*. Metabolic profiling confirmed the expected increase in glutamine levels in the *glu1-2 *mutant but revealed also changes in the levels of other amino acids.

Photosynthesis and related pathways are overall downregulated, which is consistent with the chlorotic phenotype of the *glu1-2 *mutant and the reduced amount of total chlorophyll measured in *gls *mutants [5]. The flavonoid biosynthesis was also revealed as being affected, although a more detailed analysis of the affected genes did not reveal major changes in expression levels, except for genes involved in the production of flavonoid glycosides.

The most pronounced effect at the transcriptomic level could however be seen on genes that are responsive to abiotic stresses and stimuli. Genes that had been described before as being responsive to cold, heat and drought could be identified as being upregulated in the *glu1-2 *mutant, mostly in leaves. Especially striking is the way that oxidative stress response genes and genes involved in detoxification of secondary metabolites are affected in the *glu1-2 *mutant.

Fd-GOGAT plays an important role in the photorespiratory cycle by participating in the reassimilation of released ammonia. Deregulation of photorespiration in the *glu1-2 *mutant may lead to a reduced elimination of excess excitation energy, and hence an imbalance in the redox status. In addition NH_4_^+ ^that accumulates due to the lack of reassimilation through the GS/GOGAT cycle may be perceived by the plant as a toxic compound, triggering a global stress response.

## Methods

### Plant material

The *A. thaliana *T-DNA insertion line SALK_019917 for *Fd-GOGAT1 *(*At5g04140*) was identified in the SALK T-DNA insertion mutant collection [121], seeds were obtained from the European Arabidopsis Stock Centre NASC [122] and homozygous mutant plants (called *glu1-2*) were obtained. The T-DNA insertion was checked by PCR amplification using a T-DNA primer and a *Fd-GOGAT1 *specific primer, and subsequent sequencing of the amplicon. *A. thaliana *ecotype Col-0 was used as wild-type control in all described assays.

### Plant growth conditions

Wild-type Col-0 and mutant *glu1-2 *seeds were surface sterilised and sown on solid *in vitro *cultivation medium consisting of 1x Murashige and Skoog basal salt mixture, 3% sucrose, 0.75% phytoagar (w/v), pH5.7. This medium contains 1650 mg ammonium nitrate/L. Seeds were stratified for 3 days at 4°C before being transferred to a controlled growth chamber under a 16 hour photoperiod (light intensity: 75 μmol.m^-2^.sec^-1^) at 21-23°C.

### Microarray analysis

For microarray experiments of wild-type and *glu1-2 *mutant plants, four biological replicates of each were processed simultaneously through the following procedure. Leaves and roots of 18 day old *in vitro *grown plantlets were harvested separately and immediately flash-frozen in liquid N_2_. Harvesting of tissue was performed two hours after onset of the 16 hour light period. The harvested tissue was stored at -80°C until further processing. Total RNA was extracted from plant tissue (300 mg roots, 500 mg leaves) using the RNeasy Plant Midi kit (Qiagen, Hilden, Germany) following the supplier's instructions. RNasin (Promega, Madison, USA) was added to the RNA to the final concentration of 1 U/μl. RNA quality was assessed by standard denaturing agarose gel electrophoresis and RNA concentration was measured with a NanoDrop ND-1000 (Nanodrop, Delaware, USA). To prepare the samples for microarray experiments the "GeneChip Expression Analysis" procedure of Affymetrix (Santa Clara, USA) was followed. Briefly, 10 μg total RNA was processed through a one-cycle cDNA synthesis procedure, the resulting double-stranded cDNA was cleaned up and submitted to the synthesis of biotin-labelled cRNA. This biotin-labelled cRNA was then cleaned up, quantified and fragmented before being hybridized to Affymetrix GeneChip Arabidopsis ATH1 genome arrays. After 16 hours of hybridization the arrays were processed through the washing and staining procedures, before being scanned and analysed. Microarray data files have been deposited in the Gene Expression Omnibus (GEO accession number: GSE20493).

### Statistical analysis of microarray data

The microarray data were preprocessed using the Robust Multichip Average (RMA) package [123] as implemented in R [124]. The data were normalized using quantile normalization, and expression measures were produced by fitting the RMA robust linear model. Differentially regulated genes were identified using moderated t-tests discussed in [125] and implemented in the Limma package for R. To adjust for the large number of hypothesis tests made, the q-value [126] associated with each p-value was also calculated. The q-value for a gene is the expected proportion of false positives one will get when calling that gene significant. For a gene to be considered significantly differentially expressed in this experiment its q-value was required to be lower than 0.01. In effect, this means controlling the false discovery rate (FDR) [127] in the experiment at a 0.01 level.

### Genome annotation

Genome annotation as given in the tables originates from Affymetrix (Santa Clara, USA). This was supplemented/updated by information provided by The Arabidopsis Information Service (TAIR; [128]) and the Salk Institute Genomic Analysis Laboratory (SIGnAL; [121]).

Biochemical pathways were assessed according to published data (see referred articles at the respective places in the text) and information provided by the AraCyc database [129], the Kyoto Encyclopedia of Genes and Genomes (KEGG; [130]) and the Expert Protein Analysis System (ExPASy; [131]).

Classification into GO terms were taken over from the software in question (e.g. GOstat, MapMan) but AmiGO [132] and the GO annotation at TAIR [128] were used for more extensive searches.

### NMR spectroscopy of total metabolites and principal component analysis (PCA)

Total metabolites were extracted from freeze-dried plant material according to Liang et al. [133] with some modifications. Fresh plant material (0.5 g) was freeze-dried and extracted with 1.5 ml mixture of KH_2_PO_4 _buffer (90 mM, pH 6.0) in D_2_O containing 0.05% TSP (trimethylsilyl propionic acid sodium salt, w/v) and methanol-*d*_4 _(1:1). The extract was vortexed for 30s, centrifuged at 4500 rpm for 10 min and filtered through glass wool. Five hundred microliters of the supernatant were taken for NMR spectroscopy analysis.

NMR spectroscopy was performed at 25°C on a Bruker DRX 600 spectrometer (Bruker, Rheinstetten, Germany) resonating at 600.13 MHz fitted with a 5 mm BBO probe. The frequency lock was done on MeOD. 1D ^1^H-NMR spectra were recorded with presaturation of the residual water resonance in the interscan delay using a standard Bruker pulse sequence (zgpr). A 90° excitation pulse was used to record 256 FID's with a spectral width of 8389 Hz averaged into 32 k data points with an acquisition time of 3.91 s. The interscan delay was 3 s. ^1^H, ^1^H-COSY NMR spectra with the same spectral width as the 1D spectra were recorded with 2048x512 data points in the F2xF1 directions. Presaturation of the residual water resonance was done in the interscan delay using a standard Bruker pulse sequence (cosyqfpr). 16 FID's were recorded for each increment in the F1 direction. A square sine bell window function was applied in both directions and zero filling in the F1 direction was applied to give the processed spectrum a resolution of 2048x2048 data points.

The NMR spectra were exported from TOPSPIN and imported into R [124] where the datasets were compiled. SpecAlign [134] was used for peak alignment of the spectra. Principal Component Analysis (PCA) was performed in R using the pls library of Mevik et al[135]. The loading plots from the analyses were used to identify the resonances that were different between the groups.

## Authors' contributions

RK, PW, TSJ, DHTT, TRS and AMB drafted the manuscript. RK and PW analysed the transcriptional data. TSJ performed statistical analysis of the transcriptional data. TRS performed NMR spectroscopy and analysed NMR results. TC performed the verification of the T-DNA insertion mutant and a preliminary microarray characterisation on a home-made 2K chip. PW, DHTT and AMB designed the study and AMB coordinated the study. All authors read and approved the final manuscript.

## Supplementary Material

Additional file 1 **Transcriptional data of *glu1-2***. Detailed lists of genes whose expression is affected in leaves and roots of 18 day old *glu1-2 *mutant plants Click here for file

Additional file 2 **GOstat analysis of *glu1-2 *transcriptional data**. GO_biological_process terms identified by GOstat [13] as being overrepresented (P = 0.01 with FDR/Benjamini correction) among the genes affected in *glu1-2 *mutant leaves and roots. Click here for file

Additional file 3 **PathExpress analysis of *glu1-2 *transcriptional data**. Identification by PathExpress [14] of affected pathways among the sets of genes whose expression is affected in the *glu1-2 *mutant. Click here for file

Additional file 4 **GeneBins analysis of glu1-2 transcriptional data**. BINs identified by GeneBins [15] as being affected in the datasets of genes that show a different expression in *glu1-2 *organs compared to wild type. Click here for file

Additional file 5 **2-oxoglutarate synthesis and TCA cycle**. Genes whose expression is affected in *glu1-2 *mutant leaves and that encode enzymes involved in 2-oxoglutarate synthesis and the TCA cycle. Click here for file

Additional file 6 **Photosynthesis and related pathways**. Genes whose expression is affected in *glu1-2 *mutant leaves and roots and that encode enzymes involved in photosynthesis related GO categories and bins as identified by GOstat [13], AmiGO [132] and MapMan [12] respectively. Click here for file

Additional file 7 **Transcription factors**. Genes encoding WRKY, C2H2, CCCH, MYB, bHLH or AP2/ERF transcription factors and that show different expression in the *glu1-2 *mutant. Click here for file

Additional file 8 **Shikimate pathway and flavonoid biosynthesis**. Genes implicated in the shikimate pathway and flavonoid biosynthesis, and that show different expression in the *glu1-2 *mutant. Click here for file

Additional file 9 **ABC transporters**. Genes whose expression is affected in the *glu1-2 *mutant and that encode ABC transporters. Click here for file

Additional file 10 **Cytochrome P450 monooxygenases**. Cytochrome P450-encoding genes whose expression is affected in the *glu1-2 *mutant. Click here for file

Additional file 11 **Glutathione S-transferases**. Genes encoding glutathione S-transferases (GSTs) and that show different expression in the *glu1-2 *mutant. Click here for file

Additional file 12 **UDP-glycosyltransferases**. Genes encoding UDP-glycosyltransferases (UGTs) and that show different expression in the *glu1-2 *mutant. Click here for file

Additional file 13 **MATE efflux family proteins**. Genes coding for multidrug and toxic compound extrusion (MATE) efflux carriers and that show different expression in the *glu1-2 *mutant. Click here for file

Additional file 14 **Comparison with catalase 2 deficient plants**. Comparison of induced transcriptional responses in catalase 2 deficient plants CAT2HP1 and *glu1-2 *mutant leaves. Click here for file
